# Lipidomics of Environmental Microbial Communities. II: Characterization Using Molecular Networking and Information Theory

**DOI:** 10.3389/fmicb.2021.659315

**Published:** 2021-07-12

**Authors:** Su Ding, Nicole J. Bale, Ellen C. Hopmans, Laura Villanueva, Milou G. I. Arts, Stefan Schouten, Jaap S. Sinninghe Damsté

**Affiliations:** ^1^Department of Marine Microbiology and Biogeochemistry, NIOZ Royal Institute for Sea Research, Texel, Netherlands; ^2^Department of Earth Sciences, Faculty of Geosciences, Utrecht University, Utrecht, Netherlands

**Keywords:** microbial membrane lipids, lipidome, Black Sea, molecular network, information theory, mass spectometry, biomarker, intact polar lipids (IPLs)

## Abstract

Structurally diverse, specialized lipids are crucial components of microbial membranes and other organelles and play essential roles in ecological functioning. The detection of such lipids in the environment can reveal not only the occurrence of specific microbes but also the physicochemical conditions to which they are adapted to. Traditionally, liquid chromatography coupled with mass spectrometry allowed for the detection of lipids based on chromatographic separation and individual peak identification, resulting in a limited data acquisition and targeting of certain lipid groups. Here, we explored a comprehensive profiling of microbial lipids throughout the water column of a marine euxinic basin (Black Sea) using ultra high-pressure liquid chromatography coupled with high-resolution tandem mass spectrometry (UHPLC-HRMS/MS). An information theory framework combined with molecular networking based on the similarity of the mass spectra of lipids enabled us to capture lipidomic diversity and specificity in the environment, identify novel lipids, differentiate microbial sources within a lipid group, and discover potential biomarkers for biogeochemical processes. The workflow presented here allows microbial ecologists and biogeochemists to process quickly and efficiently vast amounts of lipidome data to understand microbial lipids characteristics in ecosystems.

## Introduction

Microorganisms play a primary role in the biochemical cycles of ecosystems. Understanding what microorganisms are doing, rather than simply assessing which microorganisms are present, is essential for understanding their role within ecosystems (Vestal and White, 1989). The structural diversity of lipids and their varied physicochemical properties reflect their wide ranging functions, such as the building blocks of membranes, energy storage, signaling, and modulating protein activity (Brügger, 2014). Microbial lipids are structurally very diverse and have proven to be of great taxonomic value (Sohlenkamp and Geiger, 2016). Indeed, certain microbial lipid classes have been used as biomarkers of specific taxonomic groups: for instance, isorenieratene for the photosynthetic green sulfur bacteria *Chlorobiaceae* (Sinninghe Damsté et al., 2001; Brocks et al., 2005), ladderane lipids for anammox bacteria (Sinninghe Damsté et al., 2002; Kuypers et al., 2003; Rush et al., 2012), and heterocyst glycolipids for filamentous N_2_ fixing cyanobacteria (Wolk et al., 1994; Bauersachs et al., 2009; Kumar et al., 2010). In addition, many microorganisms regulate their membrane lipid composition in order to adapt to environmental stress, thus these lipids have the potential to be used as biomarkers for specific environmental conditions (e.g., Benning et al., 1995; Van Mooy et al., 2009; Geiger et al., 2010; Martin et al., 2011; Popendorf et al., 2011b; Elling et al., 2015).

With the development over recent decades of LC-MS methods for intact polar lipid (IPL) analysis in the environment (Sturt et al., 2004), their distributions have been reported in many marine settings (e.g., Rossel et al., 2008; Schubotz et al., 2009, 2018; Van Mooy and Fredricks, 2010; Popendorf et al., 2011a, b; Wakeham et al., 2012; Sollai et al., 2019), lakes (Ertefai et al., 2008; Bale et al., 2016, 2019) and soils (Liu et al., 2010; Rethemeyer et al., 2010; Peterse et al., 2011; Ding et al., 2020; Warren, 2020). Most of these studies either used a targeted MS/MS approach, which focused on the dominant lipids or performed MS/MS interpretation manually, based on knowledge of the fragmentation patterns of specific lipid classes. This system of data analysis is laborious, time consuming, and is restricted to a few selected spectra that can be annotated among thousands of collected spectra (Kang et al., 2020). The requirement of high-level MS expertise makes this field difficult for non-chemists to get involved in, which has been seen to preclude the scientific maturation of the field (Wakeham and Lee, 2019).

Recent advances in the field of lipidomics, using non-targeted approaches combing with computational methods, allows for comprehensive lipidome profiling without *a priori* expert MS fragmentation knowledge (Pluskal et al., 2010; Shevchenko and Simons, 2010; Han et al., 2012; Wörmer et al., 2014; Yang and Han, 2016; Collins et al., 2016; Law and Zhang, 2019; Steen et al., 2020). The combination of analytical and computational advances provides a holistic picture of the lipidome and makes it more accessible, for example for studies of microbial ecology. Information theory, a mathematical analysis of information that has been used in a broad scope of microbiome diversity (Eren et al., 2014) and transcriptome diversity (Martínez and Reyes-Valdés, 2008), has been recently applied to the resolution of UHPLC-MS/MS derived metabolomic data in plants (Li et al., 2016, 2020). Application of information theory to lipidomic data may also allow us to characterize lipidomic diversity and specificity in the environment.

Another approach, molecular networking, is an excellent tool for visualization and annotation of non-targeted mass spectra without the need for cross referencing against known spectra (Watrous et al., 2012; Wang et al., 2016; Nothias et al., 2020). In this analysis, molecules related to each other based on the similarity of their fragmentation patterns form a molecular network, resulting in automated identification of analogs and related compounds. In addition to this, molecular networking has the advantage of further clustering lipids after chromatography separation, in regard to both their headgroups and their core moieties, as its principle is based on their MS/MS fragments similarity but not on their polarity or hydrophobicity. The related lipids in a cluster often differ only marginally structurally, by simple transformations such as alkylation, unsaturation, and glycosylation (Nothias et al., 2020). In addition, improved data visualization using molecular networks allows for the discovery of unknown molecules and reveal not only their molecular diversity but also their potential biological relationships (Watrous et al., 2012; Winnikoff et al., 2014; Hartmann et al., 2017). Recently, a few studies have applied molecular networking to the natural environment, based on lipidomic or metabolomic data (Kharbush et al., 2016; Petras et al., 2017; Petras et al., 2021). The methodology applied in such studies has the potential to be applied to lipid biomarker research, and in particular to IPLs.

In our companion paper (Bale et al., 2021), we presented a method that uses two-way hierarchical clustering to visualize a large UHPLC-HRMS dataset, made up of MS^1^ spectra which had been extracted using MZmine software. This data analysis method provided an overview of the variability within a complex environmental lipidome from an euxinic marine basin (the Black Sea), without bias towards known or abundant components. However, the approach of Bale et al. (2021) does not include automated extraction of MS^2^ spectra, and hence component identification was carried out using traditional (manual) methods. Here, we use the same UHPLC-HRMS/MS dataset, but after extraction of both MS^1^ and MS^2^ spectra a combination of information theory and molecular networking was applied to group components by similarity in their structure, rather than by similarity in their depth profile (as per Bale et al., 2021). In doing so, we allow for rapid component identification, based on similarity to known lipids. This approach provides a complementary information to previous work (Wakeham et al., 2007; Schubotz et al., 2009; Sollai et al., 2019) and extracts more detailed lipidomic information, valuable for a better understanding of complex environmental lipidomes of microbial communities.

## Materials and Methods

### Sampling, Extraction, and UHPLC-HRMS/MS Analysis

A detailed description of sample collection, extraction and analysis is given in Bale et al. (2021). Briefly, suspended particulate matter (SPM) at various water depth in the water column [50–2,000 meter below sea level (mbsl)] was collected in 2013 during the PHOXY cruise (June–July 2013) in the Black Sea (Kraal et al., 2017; Sollai et al., 2019) from the PHOX2 sampling station located at 42°53.8’N, 30°40.7’E in the center of the western gyre of the Black Sea. SPM was collected on pre-ashed 142-mm-diameter 0.7-μm pore size glass fiber GF/F filters (Pall Corporation, Port Washington, NY), mounted on McLane WTS-LV *in situ* pumps (McLane Laboratories Inc., Falmouth). The filters were immediately stored at –80°C until extraction.

Freeze-dried filters were extracted using a modified Bligh-Dyer procedure. After extraction, the extracts were analyzed using Agilent 1290 Infinity I UHPLC coupled to a Q Exactive Orbitrap MS (Thermo Fisher Scientific, Waltham, MA). The output data files (^∗^.raw files) generated by the UHPLC-HRMS analyses were converted to ^∗^.mzXML files using MSconvert software. In addition to the method described in our companion paper (Bale et al., 2021), both MS^1^ and MS^2^ spectra were extracted using MZmine software (Pluskal et al., 2010) for subsequent data processing. Process steps included mass peak detection, chromatogram building and deconvolution, isotope grouping, feature alignment and gap filling (Wang et al., 2016). The absolute abundance of components was obtained after processing. Due to our extraction and analytical methods, and based on annotation by the GNPS library (see later), we expect most of the components from the molecular network we generated to be lipids, thus we used the term “lipidome” for parts of the discussion where the components are discussed.

### Creation of Molecular Networks

The combined dataset of MS/MS spectra were analyzed through The Global Natural Product Social Molecular Networking (GNPS) platform (Wang et al., 2016) using the Feature Based Molecular Networking tool (Nothias et al., 2020) to build molecular networks of the detected components in the dataset. Details can be found online at https://ccms-ucsd.github.io/GNPSDocumentation. The MS/MS dataset was filtered to remove [M+H]^+^ if the [M+NH_4_]^+^ was more abundant and vice versa, by removing all MS/MS fragment ions within ± 17 Daltons (Da) of the precursor mass-to-charge ratio (*m/z*). MS/MS spectra were divided in 50 Da windows and only the top 6 fragment ions in each 50 Da window were used. The precursor ion mass tolerance was set to 0.02 Da and the MS/MS fragment ion tolerance of 0.02 Da. A molecular network was then created where edges were filtered to have a cosine score above 0.6 (an edge with a cosine score 1.0 means two nodes are identical). Each node was connected to a maximum of 6 analogs in the network. Meanwhile, consensus spectra were searched against the GNPS spectral library with maximum analog mass difference of *m/z* 100. Precursor mass deviation and matching score (cosine) can be found online https://gnps.ucsd.edu/ProteoSAFe/status.jsp?task=871685198b1949e2a46e0e471400cdce. Molecular networks were visualized using Cytoscape 3.7.2 (Shannon et al., 2003; Smoot et al., 2010). Peak area/intensity of each MS/MS spectra was added as a metadata for the lipidome visualization across the water column of the Black Sea sample set (50–2,000 mbsl).

### Information Theory Framework

Lipids were characterized by their own unique MS/MS spectrum and relative frequency of occurrence across the water column. The Hj index, estimating lipid diversity in different samples (depths), was calculated using Shannon entropy of MS/MS (lipid species) frequency distribution derived from the abundance of MS/MS precursors by the following equation as described by Martínez and Reyes-Valdés (2008) and Li et al. (2016).

$$H_{j}=-\sum_{i=1}^{m}P_{ij}log_{2}\left(P_{ij}\right)$$

where *P*_*ij*_ correspond to relative frequency of the *i*th MS/MS (*i* = 1, 2, …, *m*) in the *j*th sample (*j* = 1, 2, …, *t*), to illustrate how abundant a specific MS/MS spectrum is relative to all others.

The average frequency of the *i*th MS/MS among samples was calculated as

$$P_{i}=\frac{1}{t}\sum_{j=1}^{t}P_{ij}$$

Individual ion components (lipid species) specificity, the S_*i*_ index, was defined as the identity of a given MS/MS regarding frequencies among all the samples. The MS/MS specificity was calculated as

$$S_{i}=\frac{1}{t}\left(\sum_{j=1}^{t}\frac{P_{ij}}{P_{i}}log_{2}\frac{P_{ij}}{P_{i}}\right)$$

Individual lipid species specificity of specific environmental sample, was defined as S_*ij*_ index.

$$S_{ij}=\sum_{j=1}^{t}\frac{P_{ij}}{P_{i}}log_{2}\frac{P_{ij}}{P_{i}}$$

The water depth lipid specificity δ_*j*_ index was measured for each *j*th sample, the average of the MS/MS specificities using the following formula

$$\delta_{j}=\sum_{i=1}^{m}P_{ij}S_{i}$$

## Results and Discussion

**FIGURE 1:**
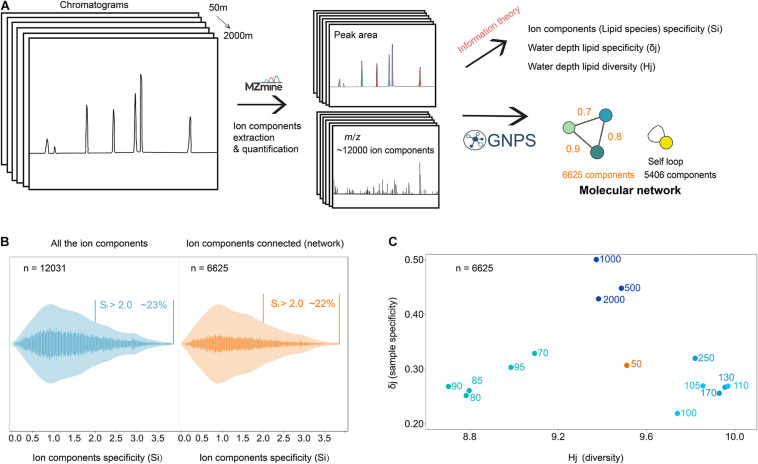
Testing the specificity and diversity of lipid species in the water column of the Black Sea using information theory and molecular network. **(A)** Schematic overview of the lipidomic data process and analysis workflow**. (B)** Specificity of the ion components among all the water column samples, left showed all the ion components while right showed the ion components connected through molecular network. Y axis represents the ion component counts, the different tones represent the trends of ion component specificity. The percentage shown here stands for the proportion of ion components that had high specificity (Si > 2), i.e., ca. 23% of all the ion components had high specificity Si > 2. **(C)** Scatter plot of Hj (diversity) vs. δj (specialization).

**FIGURE 2:**
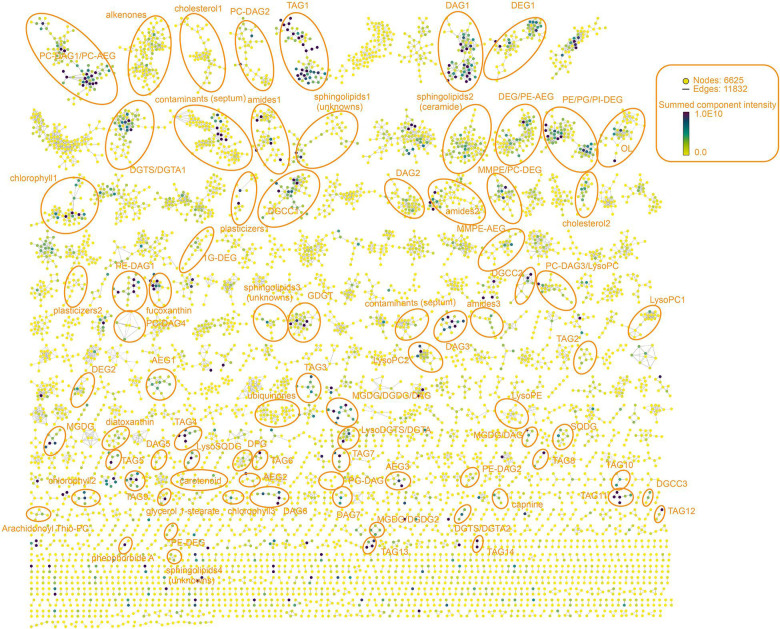
Molecular network of the water column in the Black Sea. Nodes represent MS/MS spectra of ion components (lipids) which are connected based on spectral similarity (cosine > 0.6). Nodes are labeled from light yellow to dark blue with the increasing of the summed intensity among all the water column samples (15 depths). The spatial orientation of the nodes in the MS/MS network (Figure 2) is randomly generated by Cytoscape (Shannon et al., 2003; Smoot et al., 2010) and does not relate to relationships between the subnetworks. Lipid classes (clusters) with the annotation are either tentatively identified in this study or have been reported by the previous studies (Schubotz et al., 2009, 2018; Van Mooy and Fredricks, 2010; Danielewicz et al., 2011; Wakeham et al., 2012; Bale et al., 2016; Kharbush et al., 2016; Becker et al., 2018b). DAG, diacylglycerol; DEG, dietherglycerol; AEG, acyletherglycerol; TAG, triacylglycerol; DGTS, diacylglycerylhydroxymethyltrimethyl-(N,N,N)-homoserine; DGCC, diacylglycerylcarboxyhydroxymethylcholine; DGTA, diacylglyceryl-hydroxymethyl)-tri-methyl-b-alanine; OL, ornithine lipid; DPG, diphosphatidylglycerol; PC, phosphatidylcholine; PE, phosphatidylethanolamine; PG, phosphatidylglycerol; PI, phosphatidylinositol; MMPE, phosphatidyl-(N)-methylethanolamine; MGDG, monoglycosyldiacylglycerol; GDGT, glycerol dialkyl glycerol tetraethers; SM, sphingomyelin; SQDG, sulfoquinovosyl diacylglycerol.

In the study of Bale et al. (2021) 14,648 UHPLC-HRMS^1^ components were extracted and quantified using Mzmine (Pluskal et al., 2010) in the Black Sea water dataset (SPM of 15 depths collected from 50 to 2,000 mbsl in 2013 and 10 depths from 2017). In this study we only used the dataset from 2013, from which 12,031 components with an associated MS/MS spectrum were extracted by MZmine. We applied two analytical and computational methodologies: (1) a molecular network based on the MS/MS spectra similarities (Wang et al., 2016; Nothias et al., 2020) and (2) information theory based on Shannon entropy of the lipidome distribution (Martínez and Reyes-Valdés, 2008; Li et al., 2020; Figure 1A). One of the benefits of this approach is that no code is needed to apply the workflow shown in Figure 1A. The molecular network (Figure 2) generated from GNPS (Nothias et al., 2020) contained 6625 components in familial groupings (55% of the total) and 5406 singletons (components without molecular relatives in the network). Familial groupings appear as subnetworks within the molecular network (cf. orange rings in Figure 2).

### Information Theory-Diversity and Specificity of Lipidome

Based on information theory (Martínez and Reyes-Valdés, 2008; Li et al., 2020), we calculated a set of previously established indices: ion components (lipid species) specificity (S_*i*_ index), Water depth lipid diversity (H_*j*_ index), and water depth lipid specificity (δ_*j*_ index). We examined the distribution of the S_*i*_ index across all lipids (Figure 1B; *n* = 12,031) and across those in subnetworks (familial groupings), assigned as lipids (*n* = 6,625). For the Black Sea SPM, the S_*i*_ specificity index highlights which lipids (*n* = 12,031) are specific for a certain depth of the water column, key for finding potential biomarkers associated with specific microbial communities or specific environmental conditions. A large number of lipids had small values of S_*i*_ (S_*i*_ < 2.0, 77% of the total), indicating they were either in low concentration or uniformly distributed throughout the water column. The distribution trend of specificity of the lipids connected through molecular network (S_*i*_ < 2.0, 78%) was similar to that of the total components. With the help of the S_*i*_ index (cf. Supplementary Table 2; Li et al., 2016), it is possible to focus on those lipids that are specific to certain water depths and thus for specific microbes and microbial niches.

The water depth lipid specificity (δ_*j*_) is measured as the average degree of uniqueness of individual lipids and thus is an indicator for the average lipid specificity at a certain depth of the water column. A high value of the H_*j*_ diversity index at a specific depth, is either the result of a large proportion of the total lipids being detected at that depth, or because the lipids at that depth are evenly distributed in abundance. Cross plotting the specialization and diversity of the Black Sea lipidome result in groupings of depths (Figure 1C). The deep, euxinic waters (anoxic and sulfidic, 500–2,000 mbsl) had the most specialized lipidome profiles (i.e., unique occurrence of the lipid pattern), accompanied with a medium extent of diversity among all the water column depths. This supports observations that distinct microbial communities inhabit the deep euxinic waters of the Black Sea compared to the surface waters (Durisch-Kaiser et al., 2005; Wakeham et al., 2007; Sollai et al., 2019; Suominen et al., 2020a, b). The signature for low diversity and low specialization observed at depths 70–95 mbsl (including 80, 85, and 90 mbsl) was in line with the low numbers and low intensity of resolved chromatographic peaks seen for these depths (data not shown). The suboxic/euxinic zone interface (100–250 mbsl) exhibited the most diverse lipidome profile and relatively low specialization (i.e., the lipids generally were not found uniquely in this zone) among all the water column depth samples. This finding is in accordance with previous studies that showed that a wide range of microbially mediated processes including anammox, metal reduction, sulfide oxidation, anaerobic methane oxidation and anoxygenic photosynthesis co-occur within the suboxic and upper euxinic zone of the Black Sea (Wakeham et al., 2007).

### Molecular Network: the Lipidome Throughout the Water Column

The MS/MS spectra search through the GNPS library resulted in 239 annotations (<2% annotation), which included ca. 20 contaminants, leaving the vast majority of components unknown. Such a low degree of annotation is consistent with other non-targeted environmental metabolomic studies (Petras et al., 2017, 2021). Lipidome annotation remains a bottleneck in lipidomic studies because public spectral databases are poorly populated. However, molecular networking enables the visualization of relationships between known and unknown lipids, based on the similarity of their MS/MS spectra. Despite the low percentage of library annotation, more than half the components clustered together in an individual network (Figure 2).

Most of the lipids that clustered together in the subnetworks were either analogs of each other with an identical head group or with a similar core, differing by simple transformations such as alkylation, unsaturation, and glycosylation (Nothias et al., 2020). Each node in a molecular network stands for an individual lipid associated with a specific MS/MS spectrum (see Supplementary Figure 1 as an example). Nodes in Figure 2 are labeled from light yellow to dark blue according to the lipid’s summed intensity (which is equated to abundance here) throughout the water column. Unknown lipids in a subnetwork were annotated if they were connected to one or two annotated lipids in the library (Supplementary Figure 2). Subnetworks of nodes without any library hits were annotated manually, either by comparison to data from previous studies (Rossel et al., 2008; Schubotz et al., 2009, 2018; Van Mooy and Fredricks, 2010; Danielewicz et al., 2011; Wakeham et al., 2012; Bale et al., 2016; Kharbush et al., 2016; Becker et al., 2018b) or putatively identified based on accurate mass and MS/MS fragmentation (Supplementary Table 1). Many of the subnetworks in our molecular network represent lipids that have been putatively identified earlier in studies of IPLs in the Black Sea water column and sediments (Wakeham et al., 2007; Schubotz et al., 2009; Becker et al., 2018b; Sollai et al., 2019). These included: diacylglycerol phosphatidylcholine (PC-DAG), diacylglycerol phosphatidylethanolamine (PE-DAG), diacylglycerol phosphatidylglycerol (PG-DAG), 1,2-diacylglyceryl-3-carboxyhydroxymethylcholine (DGCC), 1,2- diacylglyceryl-3-trimethylhomoserine (DGTS), diacylglyceryl-O-hydroxymethyl-(N,N,N-trimethyl)-β-alanine (DGTA), ornithine lipids (OL), monoglycosyldiacylglycerol (MGDG), dietherglycerol phosphatidylethanolamine (PE-DEG), glycosidic ceramides, isoprenoidal quinones, and glycerol dialkyl glycerol tetraethers (GDGT). Subnetworks tentatively identified within our molecular network (Figure 2) which were not reported in previous studies, included triacylglycerols (TAGs), some novel sphingolipids, certain DEG-based IPLs, and some specific chlorophylls. Our results demonstrate that a significant benefit of molecular networking is that a wider range of annotated lipids is produced with increased speed than arising by traditional analysis of LC-MS data. Molecular networking enables the grouping of different lipid classes both based on their polar head group and their core moiety. For example, there were four subnetworks of PC-DAGs (Figure 2). Some contained two short acyl carbon chain length (in sum C_28_ to C_34_) with 0–2 double bonds (Supplementary Table 1), most likely produced by algae or nitrogen–fixing or heterotrophic bacteria (Kato et al., 1996; Martínez-Morales et al., 2003; Sohlenkamp et al., 2003; Schubotz et al., 2009). The other PC-DAGs contained longer acyl carbon chain lengths (C_34_ to C_40_) with 0–8 double bonds, which are typically associated with algae (Kato et al., 1996).

**FIGURE 3:**
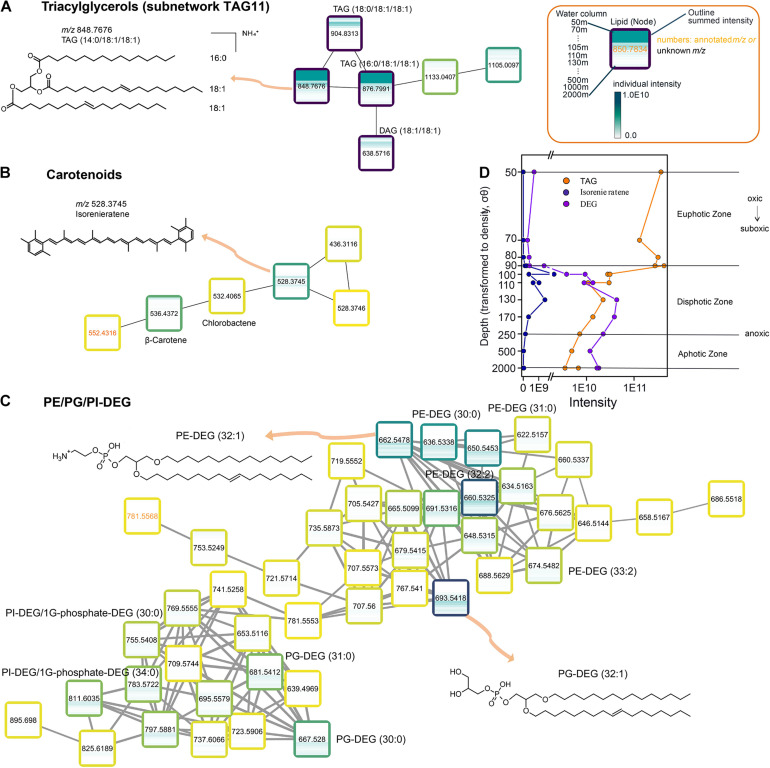
Molecular subnetwork of specific lipid classes determines the photic and aerobic zone in the Black Sea. **(A)** Subnetwork of triacylglycerol 11. **(B)** Part of subnetwork of carotenoid. **(C)** Subnetwork of PE/PG/PI-DEG. **(D)** Distribution and stratification of the summed intensity of TAG, isorenieratene and DEG in the water column of the Black Sea (50–2,000 mbsl). The names of these selected lipid classes are linked to the ones in the major network (Figure 2). Boxes shown here are the same as nodes shown in Figure 2, representing lipid species. The outlines of the boxes are labeled from light yellow to dark blue with the increasing of the summed intensity among all the water column samples (15 depths). Inside the box, a heatmap represents the distribution of this lipid species across the water column, from top (50 mbsl) to bottom (2,000 mbsl). The lipid components with closely related masses (e.g., m/z 707.5600 vs. 707.5573, **C**) have similar mass spectra but different retention time (20.9 min vs. 20.3 min), suggesting they are structural isomers.

In order to demonstrate how the molecular network can be used to understand lipidomic data in an environmental context, we discuss the depth distribution of three subnetworks: those of TAGs, carotenoids, and DEGs (Figures 3A–C). For this, the shape of each node was changed from a solid circle (as per Figure 2) to a heatmap (Figure 3), which represents the abundance gradient of the lipid from surface water to deep anoxic zone (50–2,000 mbsl).

TAGs are mostly known as energy storage lipids produced mainly by algal species under environmental stress, such as photo-oxidative stress or nitrogen deprivation (Thompson, 1996; Bigogno et al., 2002; Hu et al., 2008). They are generally synthesized in the light and then reutilized for the synthesis of other lipids in the dark (Thompson, 1996; Becker et al., 2018a), thus can be used as a marker for the euphotic and (sub)oxic zone (Figures 3A,D). The subnetwork TAG11 (Figure 3A) contains three TAGs (*m/z* 848.7676–904.8313; Supplementary Table 1), one DAG (*m/z* 638.5716, 18:1/18:1) and two unknown lipids. All these three TAGs contain a same DAG group (18:1/18:1) but differ in the third fatty acid chain length (C_14_, C_16_ or C_18_). They were present in the lower photic zone (50–90 mbsl, Figure 3A), below 90 mbsl their relative abundance decreased strikingly, by more than one order of magnitude.

The subnetwork B (Figure 3B) contains several carotenoids, including β-carotene (*m/z* 536.4372), chlorobactene (*m/z* 532.4065) and isorenieratene (*m/z* 528.3745). Isorenieratene is a carotenoid uniquely biosynthesized by the low-light-adapted photosynthetic green sulfur bacteria *Chlorobiaceae* (Repeta et al., 1989; Sinninghe Damsté et al., 1993; Sinninghe Damsté et al., 2001). *Chlorobiaceae* perform photosynthesis using sulfide, thus they require an euxinic, stratified water column with strictly anaerobic conditions and hence isorenieratene and its derivatives can be used as biomarkers for photic zone euxinia (Koopmans et al., 1996). Isorenieratene concentration was highest between 100 and 130 mbsl (Figures 3B,D), which is consistent with previous studies showing that green sulfur bacteria were present in the chemocline of the Black Sea (Overmann et al., 1992; Manske et al., 2005). Structurally closely related carotenoids such as chlorobactene and β-carotene are also found in this cluster, although with different depth distributions.

The subnetwork C (Figure 3C) contain diether glycerols (DEGs) with several types of head groups (PE/PG/PI). They differ from each other in chain length (C_30_ to C_34_) and degree of unsaturation (zero up to two). DEGs with different head groups (PE/PG/PI) have been commonly associated with sulfate-reducing bacteria (Sturt et al., 2004; Schubotz et al., 2009) and consequently are applied as indicators for anaerobic/sulfidic conditions. In our dataset, DEGs were firstly encountered in the upper anoxic zone with the appearance of sulfide (95 mbsl) and reached a maximum at 130 mbsl (Figures 3C,D).

The stratification highlighted using these three subnetworks is in agreement with the results achieved by hierarchical clustering of the same MZmine data by Bale et al. (2021). It shows that applying molecular networking to the water column lipidome has the potential to deliver biologically meaningful lipid-microbial community associations or environmental factors on lipid distribution.

### Different Microbial Sources Within the Same Lipid Class

**FIGURE 4:**
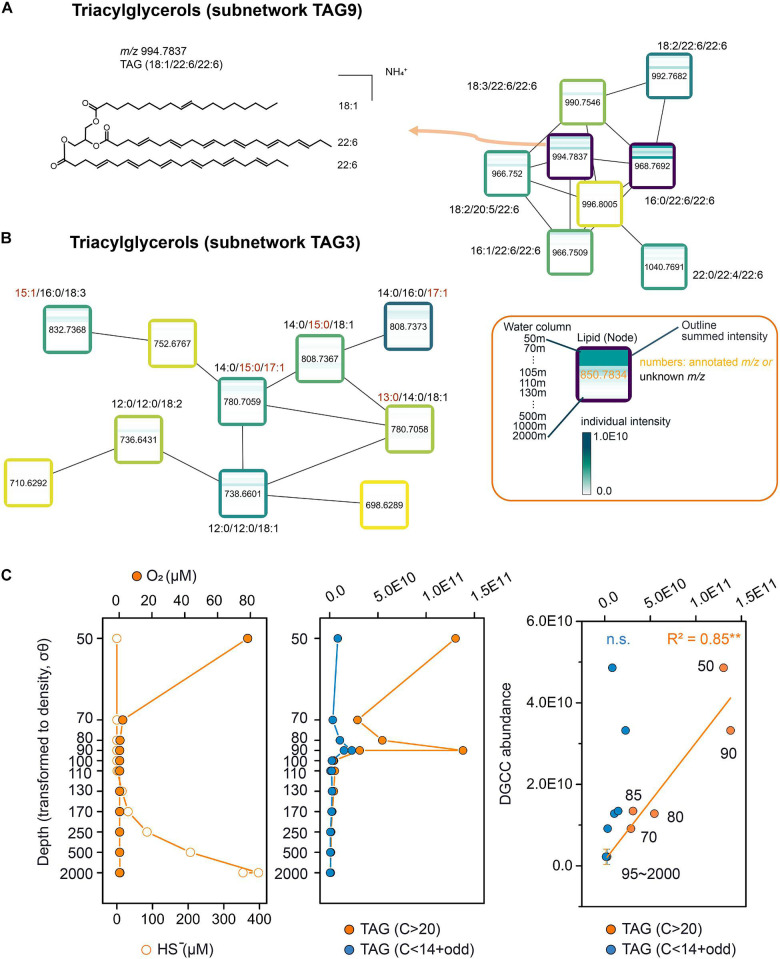
Molecular subnetwork of triacylglycerols and their source determination. **(A)** Subnetwork of TAG9**. (B)** Subnetwork of TAG3. **(C)** From left to right: Distribution of oxygen (O_2_, μM, orange solid cycles) and sulfide (HS^–^, μM, orange hollow cycles) in the water column of the Black Sea (50–2,000 mbsl); Distribution of summed TAGs with polyunsaturated fatty acids (at least one of the three acyl chain length with more than C_20_, orange cycles) and summed TAGs with odd (C_15_ and C_17_) and short (C_12_ and C_14_) carbon chain length (blue cycles); Scatter plots of DGCC vs. TAGs with polyunsaturated fatty acids (orange cycles) and TAGs with odd (C_15_ and C_17_) and short (C_12_ and C_14_) carbon chain length (blue cycles). Significance: **P < 0.01; n.s., not significant. The names of these selected lipid classes are linked to the ones in the major network (Figure 2). Boxes shown in subnetwork are the same as nodes shown in Figure 2, representing lipid species. The outlines of the boxes are labeled from light yellow to dark blue with the increasing of the summed intensity among all the water column samples (15 depths). Inside the box, a heatmap represents the distribution of this lipid species across the water column, from top (50 mbsl) to bottom (2,000 mbsl).

Molecular networking enables us to investigate the potential sources of individual groups of compounds in the same polar lipid classes. For example, there were fourteen separate subnetworks of TAGs (Figure 2), indicating that groups of TAGs differed significantly from each other in carbon chain length or degree of unsaturation. TAGs which contain a polyunsaturated fatty acid (FA; e.g., TAG-C_16:0_/C_22:6_/C_22:6;_ clustered in TAG9) were predominant in the euphotic zone (50–90 m; Figure 4A). Algal species such as *Euglenophyceae*, *Cryptophyceae*, and *Eustigmatophyceae* possess the ability to synthesize these TAGs (Hu et al., 2008). Similar depth distributions were also detected in the subnetwork of TAG11 (Figure 3A). These TAGs contain a FA with carbon chain lengths of C_16_ and C_18_ and 0–2 double bonds (Figure 3A), they are also known as typical storage lipids in most algae (Volkman et al., 1989). Distinct from the subnetworks of TAGs mentioned above, subnetworks of TAGs with odd (C_15_ and C_17_; likely *iso*) and short (C_12_ and C_14_) acyl chain length, were only shown to be abundant at 90 mbsl (TAG3, Figure 4B), suggesting they have a different source. To further determine the source of different types of TAGs mentioned above, we compared betaine DGCC lipids to the long-chain polyunsaturated TAGs (which contain at least one acyl chain with > 20 carbon atoms) and short-odd-chain TAGs (which contain C_12_, C_14_, C_15_, or C_17_ acyl chains). DGCC lipids are supposed to be produced mainly by algae (Kato et al., 1994; Van Mooy et al., 2009). We found there was no correlation between the latter TAGs and DGCC lipids, while the DGCCs did correlate with TAGs containing even carbon numbered, long-chain polyunsaturated fatty acids (*R*^2^ = 0.85, *P* < 0.01; Figure 4C). This suggests non-algae sources for the TAGs with short and odd chain fatty acids, such as the bacteria actinomycetes which are capable of producing odd chain TAGs (Alvarez and Steinbüchel, 2002).

**FIGURE 5:**
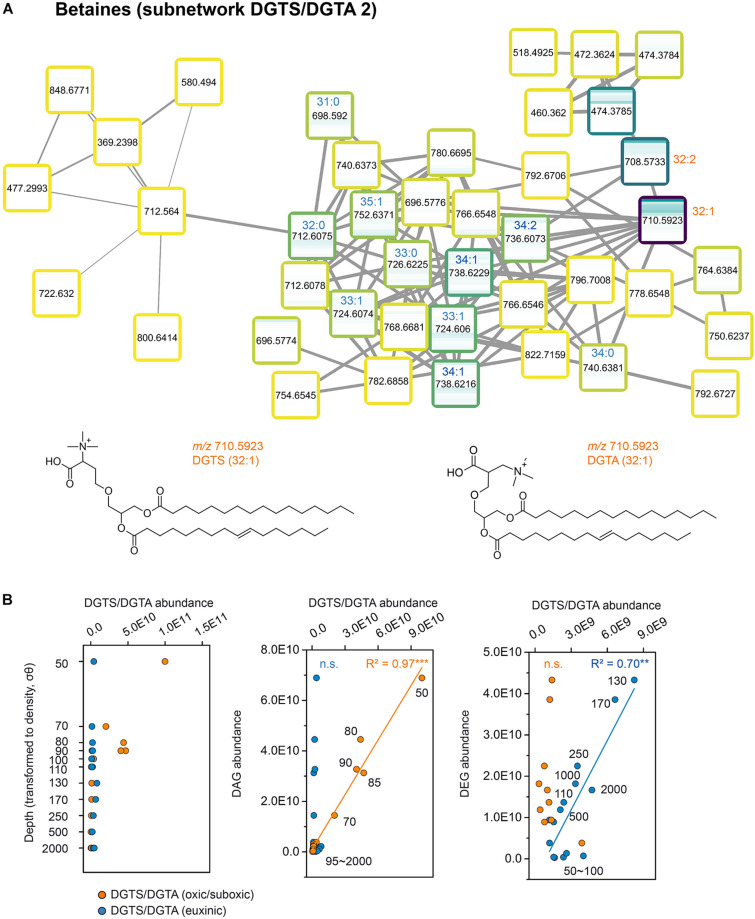
Molecular subnetwork of betaine lipids (DGTS/DGTA) and their source determination. **(A)** Subnetwork of betaine lipids DGTS/DGTA. **(B)** From left to right: Distribution of DGTS/DGTA (oxic to suboxic, abundant at the surface water 50–90 mbsl, including DGTS/DGTA with C_32:2_, C_32:1_, C_34_ to C_40_ carbon chain length and 0–10 double bonds, orange cycles) and DGTS/DGTA(euxinic, abundant at the deep water 110–2,000 mbsl, including DGTS/DGTA with C_31_ to C_35_ carbon chain length and 0–2 double bonds, blue cycles); Scatter plots of DAG vs. DGTS/DGTA (oxic/suboxic, orange cycles) and DGTS/DGTA (euxinic, blue cycles); Scatter plots of DEG with several head groups (PE/PG/PI) vs. DGTS/DGTA (oxic/suboxic, orange cycles) and DGTS/DGTA (euxinic, blue cycles). Significance: ***P < 0.001; **P < 0.01; n.s., not significant. The name of selected DGTS/DGTA is linked to the one in the major network (Figure 2). Boxes shown in subnetwork are the same as nodes shown in Figure 2, representing lipid species. The outlines of the boxes are labeled from light yellow to dark blue with the increasing of the summed intensity among all the water column samples (15 depths). Inside the box, a heatmap represents the distribution of this lipid species across the water column, from top (50 mbsl) to bottom (2,000 mbsl).

Another distinct depth pattern was found in the subnetwork of the betaine lipids DGTS and DGTA (Figure 5). DGTS and DGTA are structural isomers with the same characteristic fragment ions in the MS/MS spectra, and hence they cluster in the same subnetwork. Since DGTS cannot be distinguished from DGTA based on mass spectra, we hereafter indicate these betaine lipids as DGTS/DGTA for the following discussion. DGTS/DGTA lipids with C_32:2_ and C_32:1_ acyl chains were dominant in the oxic zone (50–90 mbsl; Figure 5A). The sum of their abundance, together with another cluster of DGTS/DGTA with longer (C_34_ to C_40_) acyl chains with 0–10 double bonds (DGTS/DGTA1 in Figure 2 and Supplementary Table 1), were significantly correlated with the abundance of headgroup-less DAGs (*R*^2^ = 0.97, *P* < 0.001; Figure 5B). DAGs were dominant in the oxic and suboxic zone and are likely catabolic products or biosynthetic intermediates of TAGs, phospholipids and glycolipids, derived mainly from algae (Kharbush et al., 2016; Becker et al., 2018a). The correlation strongly suggesting that these DGTS/DGTA lipids were also derived from algae (Dembitsky, 1996; Van Mooy and Fredricks, 2010). A different subnetwork of DGTS/DGTA with C_31_ to C_35_ acyl chains and 0–2 double bonds were absent from the surface waters but were relatively more abundant in deep suboxic and euxinic zone (110–2,000 mbsl) with a peak at 130 mbsl (Figure 5A). Significant correlation was found between the intensity of these DGTS/DGTA lipids with that of DEGs with and without headgroups (*R*^2^ = 0.70, *P* < 0.01; Figure 5B). In contrast to DAGs, DEGs in the Black Sea water column has been shown to correlate with sulfate reducing bacteria (Neretin et al., 2007) and in general are mostly associated with anaerobic bacteria (Grossi et al., 2015). This suggests these types of betaine lipids were derived from anaerobic bacteria such as sulfate reducing bacteria (López-Lara et al., 2003) or other bacteria residing in the euxinic zone. Although distinct patterns of DGTS/DGTA lipids throughout the water column were also observed in previous studies (Schubotz et al., 2009; Van Mooy and Fredricks, 2010), here the application of molecular networking enables the direct observation of individual DGTS/DGTA distributions across the water column.

### Targeting Unknown Lipids

Given the diversity and complexity of lipids from environmental samples, less abundant but perhaps ecologically informative lipids cannot be easily detected if they coelute with more dominant components of the lipidome. With the help of a molecular network, embedded with the heatmaps of the lipids’ variation across the ecosystem, one can rapidly pick out significant unknown lipids at certain depths or specific environmental conditions. A great number of such lipids can then be putatively identified by comparing their MS/MS fragmentation pattern to the associated ones if they are in a subnetwork.

**FIGURE 6:**
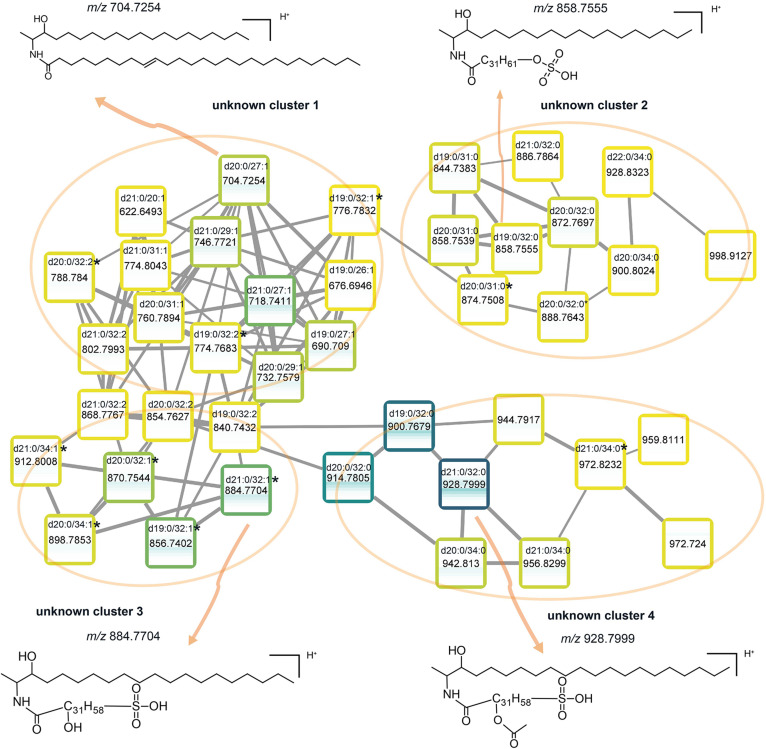
Molecular subnetwork of sphingolipids1. Boxes shown in subnetwork are the same as nodes shown in Figure 2, representing lipid species. The outlines of the boxes are labeled from light yellow to dark blue with the increasing of the summed intensity among all the water column samples (15 depths). Inside the box, a heatmap represents the distribution of this lipid species across the water column, from top (50 mbsl) to bottom (2,000 mbsl). ^∗^Represents there is a –OH group in the fatty acid chain of the lipid. Mass spectra of novel lipids are shown in Supplementary Figures 3A–D.

Many of the subnetworks in our dataset (Figure 2) contained unknown components. The MS/MS pattern recognition, intrinsic to the molecular network data processing, provide an indication as to their structures, based on similarity to known lipids within the same subnetwork. To illustrate this, we focus first on a subnetwork (Sphingolipids1) that contains four cluster of unassigned lipids (Figure 6). From a first cursory inspection of the MS/MS mass spectra associated with these components (Supplementary Figures 3A–D), it became clear that all these unassigned components shared structural features, explaining why these clusters are connected, but differed from each other in complexity of the whole molecule (see Supplementary Text and Supplementary Figure 3 for details). The first unknown cluster (Figure 6, cluster 1) contains 13 lipid species. The MS/MS spectrum from one of the members of this cluster (*m/z* 704.7254, Figure 6 and Supplementary Figure 2A) was very similar to that of a ceramide standard (d18:1/24:0, *m/z* 650.6454, Supplementary Figure 4), but with one less loss of H_2_O in the unknown lipid. We therefore tentatively identify it as 1-deoxyceramide (d20:0/27:1). Other lipid species in the cluster 1 are also 1-deoxyceramides, differing from each other in the chain lengths of 1-deoxysphinganine base (C_19_ to C_21_, Figure 6) and/or fatty acid groups (C_20_ to C_32_).

1-deoxysphinganine, the sphingoid base of the 1-deoxyceramides was first found in a marine organism, *Spisula polynyma* (Cuadros et al., 2000). Since then, 1-deoxyceramides were typically reported as “total 1-deoxysphinganines” because they were quantified after acid hydrolysis to release the sphingoid bases (Merrill, 2011). With the technical progress in HPLC-HRMS over the last few years, it is now possible to analyze 1-deoxycermide as individual molecular species (Duan and Merrill, 2015). These atypical headless sphingolipids cannot be degraded over the canonical catabolic pathways and are incapable to be converted to complex sphingolipids (Lone et al., 2019). Due to the lack of the C1-OH, they cannot be further metabolized into more complex sphingolipids and the understanding of their biological functions is still limited (Carreira et al., 2019).

The other three clusters of unknown components that connected to the cluster 1 are 1-deoxyceramides with polar moieties (Figure 6). Unlike the common sphingolipids which contain polar moieties as “headgroups” at the C1-OH position (Walker et al., 2017; Heaver et al., 2018), the polar moieties of these unusual 1-deoxyceramides appear to be located on their fatty acid chain (Supplementary Text and Supplementary Figures 3B–D). Based on the MS/MS spectrum, the lipid species in the second unknown cluster contain a sulfate moiety on their fatty acid chain, thus they are tentatively identified as sulfate-1-deoxyceramides (Supplementary Figure 3B). The third unknown cluster consists of 1-deoxyceramides containing a hydroxy-fatty acid modified with a sulfur trioxide moiety (Supplementary Figure 3C). We therefore proposed them as sulfono-1-deoxyceramides. The fourth and last unknown cluster has 1-deoxyceramides with an extra acetic acid and a sulfur trioxide moiety on their fatty acid chain (Supplementary Figure 3D), hence, we assigned these components as acetylsulfono-1-deoxyceramides.

All these newly putatively identified sphingolipids were at their maximum abundance at the interface between the suboxic and euxinic zones (95–250 mbsl; Figure 6). Bacterial sphingolipids are phylogenetically restricted to be produced by mainly members of the *Bacteroidetes* and selected *Proteobacteria* (Heaver et al., 2018). Certain *Bacteroidetes* are known to produce capnines, sulfono-analogs of sphinganines (Godchaux III, and Leadbetter, 1980). Recently three members of the *Bacteroidetes*, *Ancylomarina euxinus* sp. nov. *Labilibaculum euxinus* sp. nov., and *Lutibacter* sp., all isolated from the euxinic zone of the Black Sea, were found to have capnines among their most abundant lipids (Bale et al., 2020; Yadav et al., 2020). Capnines were also found in the molecular network (Figure 2). We hypothesize that the novel 1-deoxysphingolipids putatively identified in this study, may also be produced by anaerobic heterotrophs related to *Bacteroidetes*.

**FIGURE 7:**
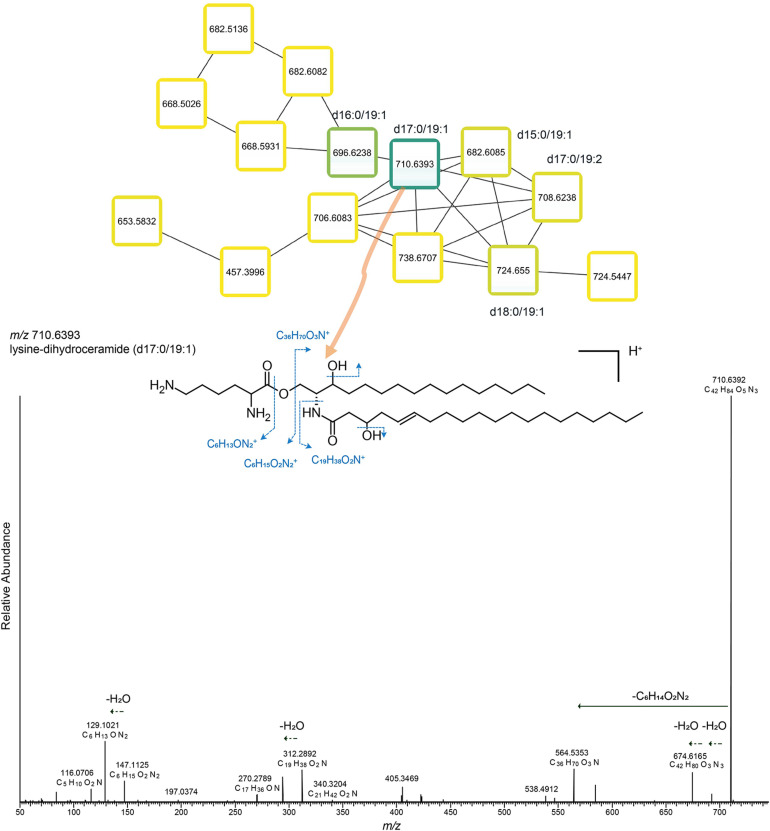
Molecular subnetwork of sphingolipids3. Boxes shown in subnetwork are the same as nodes shown in Figure 2, representing lipid species. The outlines of the boxes are labeled from light yellow to dark blue with the increasing of the summed intensity among all the water column samples (15 depths). Inside the box, a heatmap represents the distribution of this lipid species across the water column, from top (50 mbsl) to bottom (2,000 mbsl).

Another example of a subnetwork is one that only contained unknown lipids (Figure 7) and which was associated within the euxinic zone (130–2,000 mbsl). All 14 lipids in the subnetwork exhibited sphingolipid-like MS/MS fragmentation, but unlike the earlier-mentioned sphingolipid subnetwork (Figure 6), these MS/MS spectra (Figure 7 and Supplementary Text) revealed that these lipids contained relatively short dehydrosphinganine bases (C_15_ to C_18_) connected to a longer chain hydroxy fatty acid (C_19_). The polar head group was tentatively identified as a lysine (Moore et al., 2016). Therefore, they were tentatively assigned as lysine-dihydroceramides (Figure 7 and Supplementary Text). To the best of our knowledge, this is the first report to indicate the presence of lysinesphingolipids in environmental samples. Their presence in the euxinic zone suggests they are derived from anaerobic bacteria. Among all the annotated lipids, the newly putatively identified sphingolipids (Figures 6, 7) have the highest S_*ij*_ index (Supplementary Table 2) in the deep zone (130–1,000 mbsl). The higher S_*ij*_ index is, the more the occurrence of a certain lipids is restricted to at certain depth. Thus, these newly putatively identified sphingolipids are one of the most specialized lipids in this distinct euxinic region.

## Conclusion

In this study, we carried out comprehensive lipidomic profiling of microbial communities throughout the water column of the Black Sea using UHPLC-HRMS/MS spectra. A major strength of our data processing method is that we combined information theory and molecular networking to capture a holistic view of the lipidome as well as specific signatures in the environment. Indeed, the molecular network provided a comprehensible visualization of the lipidome throughout the water column, while information theory allowed us to capture the signatures of diversity and specialization within the lipidome. Application of molecular networking has proven to be useful in discovering novel lipids, helping to determine their origin, and associating biomarkers with potential microbial niches. Another advantage on this method is that the diversity of unknown lipids is revealed before they are identified. In conclusion, this study reinforces a powerful set of computational approaches to accelerate our understanding of lipidomic information in environmental microbial ecology.

## Data Availability Statement

The raw data supporting the conclusions of this article will be made available by the authors, without undue reservation.

## Author Contributions

SD, NB, EH, LV, SS, and JSSD designed the study. SD performed the data analysis. SD, NB, and EH did the lipid identification and wrote the draft manuscript. MGIA provided methodological support. SS and JSSD supervised the study. All authors read, discussed, and approved the final manuscript.

## Conflict of Interest

The authors declare that the research was conducted in the absence of any commercial or financial relationships that could be construed as a potential conflict of interest.
