# Supplementary material for: Lipidomics of Environmental Microbial Communities. II: Characterization Using Molecular Networking and Information Theory
Source: Front Microbiol. 2021 Jul 12;12:659315. doi: 10.3389/fmicb.2021.659315 (PMC8311935; doi:10.3389/fmicb.2021.659315)
Supplement: Supplementary file 1 [file Data_Sheet_1.docx]

**Lipidomics of environmental microbial communities. II: Characterization using molecular networking and information theory**

Su Ding^1^, Nicole J. Bale^1^, Ellen C. Hopmans^1^, Laura Villanueva^1,2^, Milou Arts^1,2^, Stefan Schouten^1,2^, Jaap S. Sinninghe Damsté^1,2^

^1^NIOZ Royal Institute for Sea Research, Department of Marine Microbiology and Biogeochemistry, Texel, The Netherlands

*^2^Department of Earth Sciences, Faculty of Geosciences, Utrecht University, Utrecht, The Netherlands*

**Supporting information**

**Supplementary Text**

**Figure S1-S4**

**Table S1-S2**

**Supplementary Text**

**Identification of clusters within unassigned subnetwork Sphingolipids1**

Many of the subnetworks in our dataset (Fig. 2) contained unassigned components. Here, to illustrate how the molecular network can lead to assignment and identification of (novel) classes of lipids we elucidated the identity of two of these networks. We focus first on a subnetwork (Sphingolipids1) that contains 4 clusters of unassigned components (Fig. 6; see Fig. S4 for representative MS^2^ spectra from each cluster). From a first cursory inspection of the MS^2^ mass spectra associated with these components, it became clear that all these unassigned components shared structural features, explaining why these clusters are connected, but differed from each other in complexity of the whole molecule.

Unknown cluster 1 is the most populated cluster (13 nodes) within the sphingolipid 1 subnetwork (Fig. 6). Fig. S3A shows the MS^2^ fragmentation spectrum of one of the members of this cluster, labeled with *m/z* 704.7270 (*m/z* 704.7254 in Fig. 6). Inspection of MS^1^ data revealed a peak at 37.60 minutes with *m/z* 704.7270 and assigned EC of C_47_H_94_O_2_N^+^ (Δm = -1.54 mmu). The MS^2^ spectrum showed an initial loss of H_2_O (Δm = -0.13 mmu) and the formation of two pairs of fragment ions separated by 18 Da (H_2_O). The most dominant pair of fragment ions had an AEC of C_20_H_42_N^+^ (*m/z* 296.3305, Δm = -0.67 mmu) and C_20_H_44_ON^+^ (*m/z* 314.3409, Δm = -0.80 mmu). In addition there is a minor fragment at *m/z* 408.4182 (EC C_27_H_54_ON^+^; Δm = -0.90 mmu). The loss of (one or) more alcohol moieties as H_2_O and formation of nitrogen containing core lipids is well described for various sphingolipids. Fig. S4 shows the MS^2^ spectrum of an authentic ceramide standard (d18:0/24:0; contained in ceramide lipidomix, Avanti Polar lipids, Alabaster, Alabama, USA), fragmented under identical conditions as used in this study. Here we do not observe the molecular ion ([M+H]^+^), but instead two fragments ions at m/z 632 and 614 are formed after 2 consecutive losses of H_2_O. Cleavage on either side of the nitrogen results in a fragment ion at *m/z* 282 (C_18_H_36_ON^+^), and after loss of a further 18 Da (H_2_O), in the base peak at *m/z* 264 (C_18_H_34_N^+^), representing the sphingoid base (in this case sphingosine). In addition, a minor fragment is observed at *m/z* 368.3886 (C_24_H_50_ON^+^) representing the fatty acid moiety. The observed fragmentation for this ceramide standard closely matches the fragmentation pattern observed for unknown cluster 1, with the exception that we observe 1 loss of 18 Da (H_2_O) from the molecular ion instead of the 2 consecutive losses observed for the standard ceramide. Based on the similarity of its MS^2^ spectrum to that of ceramide, but with one less loss of H_2_O, we tentatively identify unknown cluster 1 as 1-deoxyceramides, and the component with *m/z* 704 as 1-deoxyceramide (d20:0/27:1) of which the structure and diagnostic fragmentations are shown in Fig. S3A. In addition to the two C_20_ fragments representing the C_20_ 1-deoxysphinganine base, a pair of C_19_ fragments is observed in the MS^2^ spectrum. Careful examination of the spectrum showed the complimentary fragment ion at *m/z* 422.4371 (AEC C_28_H_56_ON^+^, Δm = -0.001 mmu). This shows that this MS^2^ spectrum, in fact, represents the mixed spectrum of 2 isobaric 1-deoxyceramides, the first being a 1- deoxyceramide (d20:0/27:1) and the second being a 1- deoxyceramide (d19:0/28:1).

The second unknown cluster within the sphingolipid 1 subnetwork is made up of ten components with *m/z* values between 844.7383 and 998.9127 (Fig. 6). Fig. S3B shows the MS^2^ spectrum of a member of this cluster with a retention time 23.57 min and *m/z* 858.7569 (*m/z* 858.7555 in Fig. 6) with an assigned EC of C_51_H_104_O_6_NS^+^ (Δm = -1.92 mmu). The molecular ion is not observed in the MS spectrum, however a dominant fragment ion at *m/z* 760.7890 with AEC of C_51_H_102_O_2_N^+^ is observed (Δm = -1.51 mmu), showing that the initial neutral of 80 Da was H_2_SO_4_ (sulfate moiety). The sulfate moiety appears to be located on the fatty acid chain, as the main fragment ions at *m/z* 282 and 300 have an AEC expected for a 1-deoxysphinganine base (C_19_H_40_N^+^ and C_19_H_40_ON^+^, respectively), while the minor fragment representing the fatty acid moiety at *m/z* 478.4968 (AEC C_32_H_64_ON^+^, Δm = -0.002 mmu) now shows an unsaturation due to the previous loss of the sulfate moiety. We therefore tentatively identify this novel sphingolipid as sulfate-1-deoxyceramide (d19:0/32:0) and unknown cluster 2 as sulfate-1-deoxyceramides. Similar as described above for the 1-deoxyceramide, the MS^2^ spectrum shown in Fig. S3B is a mixed spectrum of 2 isobaric species as fragments indicating a d21:0/30:0 species are also present.

The third unknown cluster within the subnetwork of sphingolipids 1 is made up of eight components with *m/z* values between 840.7432 and 912.8008, of which the MS^2^ of a member at 23.22 min with *m/z* 884.7718 (*m/z* 884.7704 in Fig. 6) and an AEC of C_53_H_106_O_6_NS^+^ (Δm = -0.95 mmu) is shown in Fig. S3C. Whereas the fragmentation of the deoxyceramide sulfates was characterized by the initial loss of H_2_SO_4_ (80 Da), here we observe an initial loss of 81.9725 Da with an assigned EC of H_2_SO_3_ (Δm = -0.07 mmu), suggesting the presence of a sulfur trioxide moiety in the molecule. The fragment ions produced from the deoxy-sphinganine base (at *m/z* 310 and 328) are as expected for a C_21_-deoxysphinganine base, thus indicating that, also in this family of molecules, the modification is on the conjugated acetic acid. The fragments ions representing the fatty acid moiety are observed at *m/z* 475.4504 (C_32_H_59_O_2_^+^, Δm = -0.52 mmu) and 492.4777 (C_32_H_62_O_2_N^+^, Δm = -0.15 mmu). The assigned EC clearly shows an additional O-containing functionality on the fatty acid chain; therefore we propose this family of sphingolipids consists of 1-deoxyceramides containing a hydroxy-fatty acid modified with a sulfur trioxide moiety. The sulfur trioxide appears to be directly linked to the fatty acid by the sulfur atom, thus creating a sulfono-1-deoxyceramide. The component shown in Fig. 6C is determined to be a sulfono-1-deoxyceramide (d21:0/32:1).

The fourth and last unknown cluster within this subnetwork of sphingolipids, connected directly to cluster 3, contains nine components with *m/z* values between 900.7679 and 972.8232. The MS^2^ spectrum of a component eluting at 23.84 min with *m/z* 928.7983 (AEC C_55_H_110_O_7_NS^+^, Δm = -1.21 mmu) is shown in Fig. S3D (*m/z* 928.7999 in Fig. 6). Upon MS^2^ fragmentation, this component undergoes an initial neutral loss of 60.0216 Da corresponding to acetic acid, (C_2_H_4_O_2,_ Δm = 0.42 mmu), followed by a loss of sulfurous acid (H_2_O_3_S), and production of fragment ions at *m/z* 310 and 324, identical to what was observed for the sulfonolipid shown in Fig. S3C. Hence, we assigned these components as acetylsulfono-1-deoxyceramides, where the component with *m/z* 928 consists of a C21:0 1-deoxysphinganine and a C32:0 acetyl-sulfono-fatty acid.

For all discussed components above, the structural identification is tentative. Further structural elucidation methods (e.g., nuclear magnetic resonance [NMR] spectroscopy) would be required to confirm the proposed structures and elucidate the position of the various functional groups.

**Identification of clusters within unassigned subnetwork sphingolipids 3**

This subnetwork consists of 14 lipids, which exhibit sphingolipid-like MS/MS fragmentation. Fig. 7 shows the MS^2^ spectrum of one of the components in this network (eluting at 16.57 min, *m/z* 710.6391, AEC C_42_H_84_O_5_N_3_^+^, Δm = 1.39 mmu). An initial neutral loss of 146.1039 (C_6_H_14_O_2_N_2_, Δm = 1.03 mmu) results in a fragment at *m/z* 564.5353 (C_36_H_70_O_3_N^+^, Δm = 0.26 mmu) and further fragmentation releases three fragment ions: a pair of ions, separated by 18 Da, at *m/z* 294.2787 and *m/z* 312.2892 with AECs of C_19_H_36_ON^+^ (Δm = -0.42 mmu) and C_19_H_38_O_2_N^+^ (Δm = -0.53 mmu), respectively, and a third ion at a *m/z* 270.2789 with an AEC of C_17_H_36_ON^+^ (Δm = -0.21 mmu). Based on these fragments we propose that the ceramide core of this lipid consists of a C17 dihydrosphingosine bound to a C19:1 hydroxy fatty acid (Fig. 7) (i.e. a dihydroceramide)

The initial neutral loss of 146 Da is associated with two fragments at *m/z* 147.1126 (C_6_H_15_N_2_O_2_^+^) and at *m/z* 129.1022 (C_6_H_13_N_2_O^+^). These fragments were earlier identified by Moore et al. (2016) as diagnostic for lysine lipids. We therefore tentatively identify this cluster of lipids as a lysine-dihydroceramides.


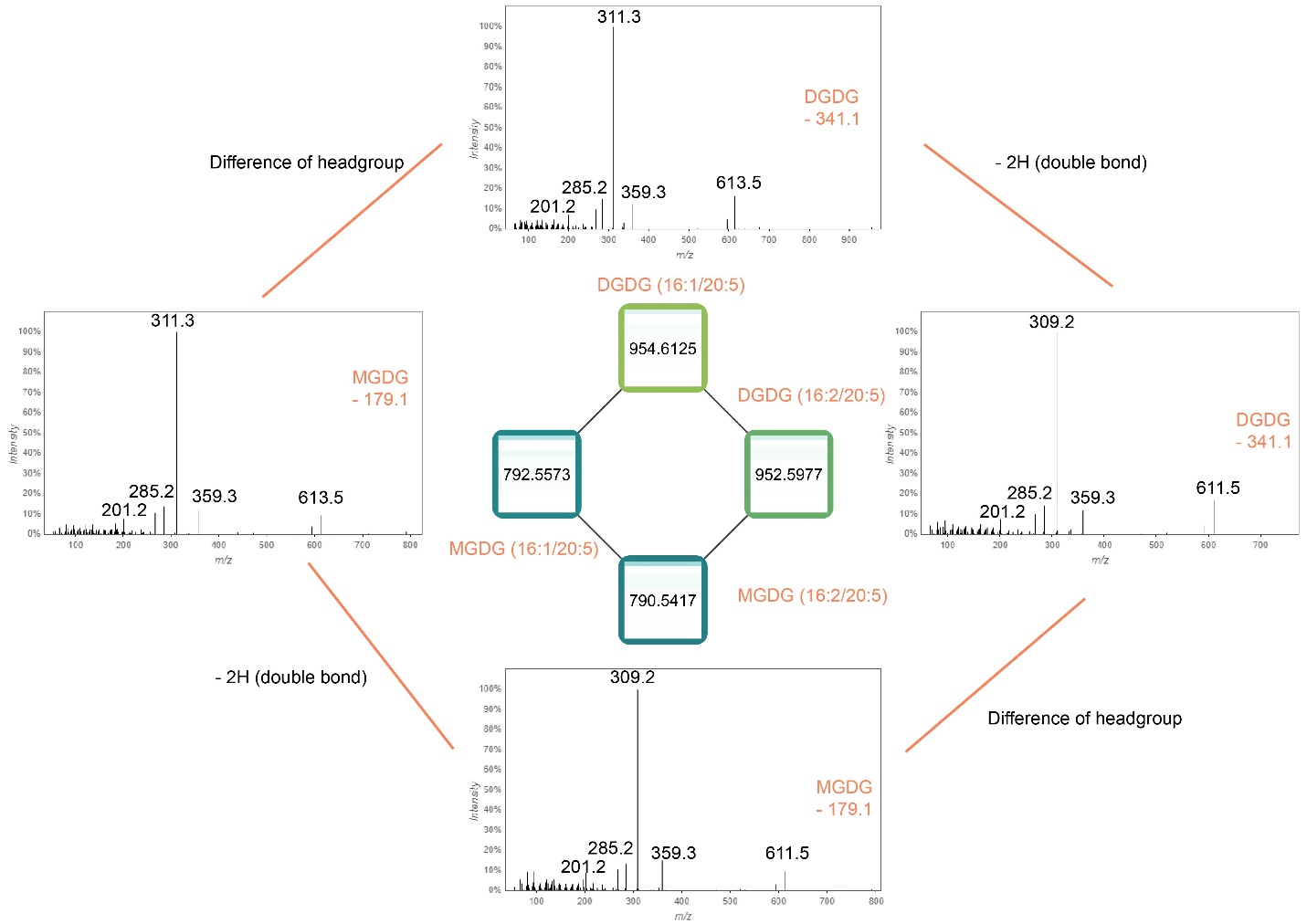


**Figure S1.** Example of lipid subnetwork of MGDG/DGDG2 (Fig. 2), showing how lipids in a cluster are correlated to each other. This includes lipids’ precursor mass, mass spectra and similarity (cosine on the edge between two lipids) of fragmentation of their mass spectra.


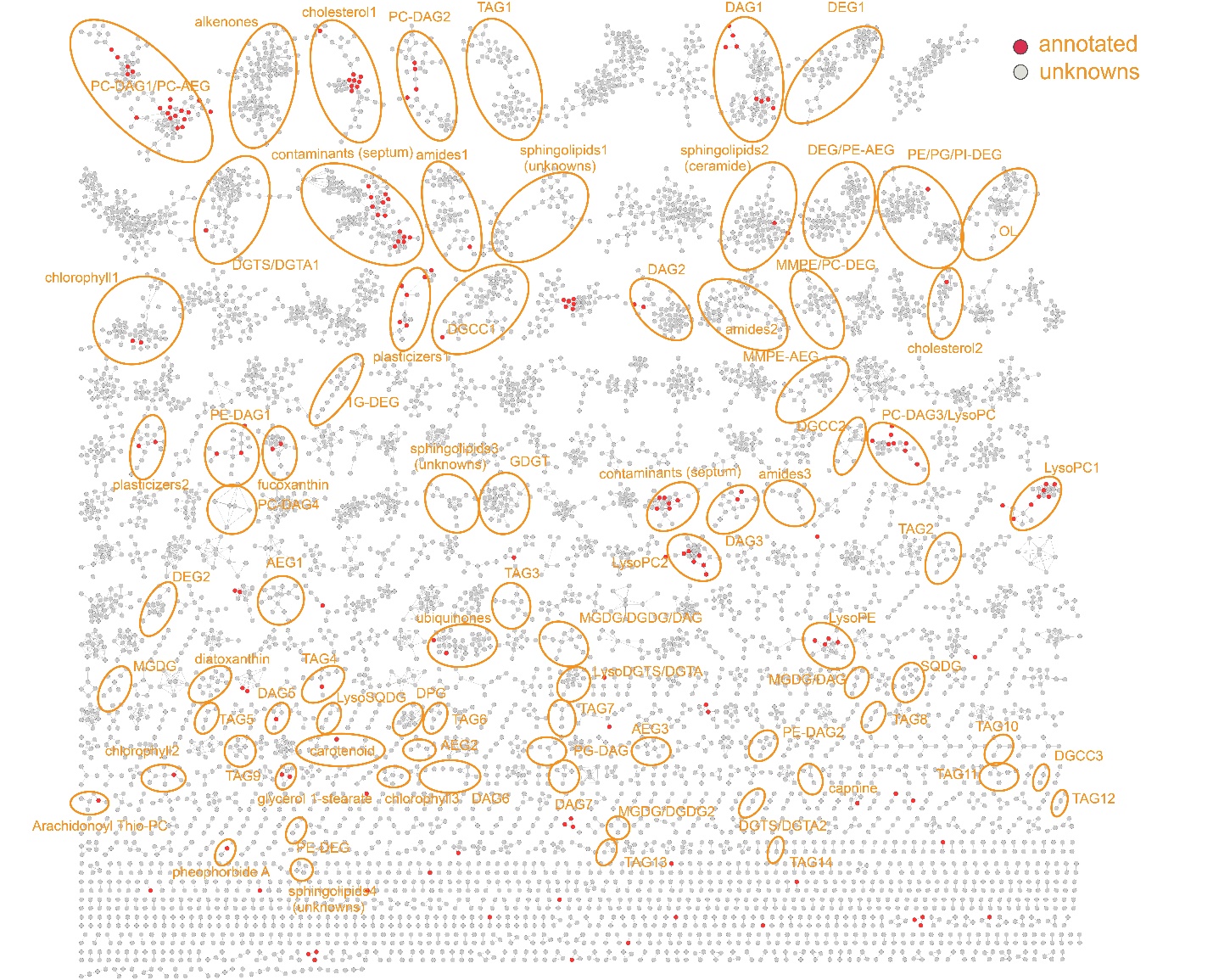


**Figure S2.** Molecular network of the water column in the Black Sea, showing annotated vs non-annotated nodes. All clusters containing an annotated node are circled. Nodes in red are annotated using the GNPS library (<https://gnps.ucsd.edu/ProteoSAFe/status.jsp?task=871685198b1949e2a46e0e471400cdce>), while nodes in gray represent compounds not found in the library. Annotated lipid classes (clusters) not containing a red node were either identified in this study or have been reported by previous studies (Schubotz et al., 2009; Van Mooy and Fredricks, 2010; Danielewicz et al., 2011; Wakeham et al., 2012; Bale et al., 2016; Kharbush et al., 2016; Becker et al., 2018b; Schubotz et al., 2018).


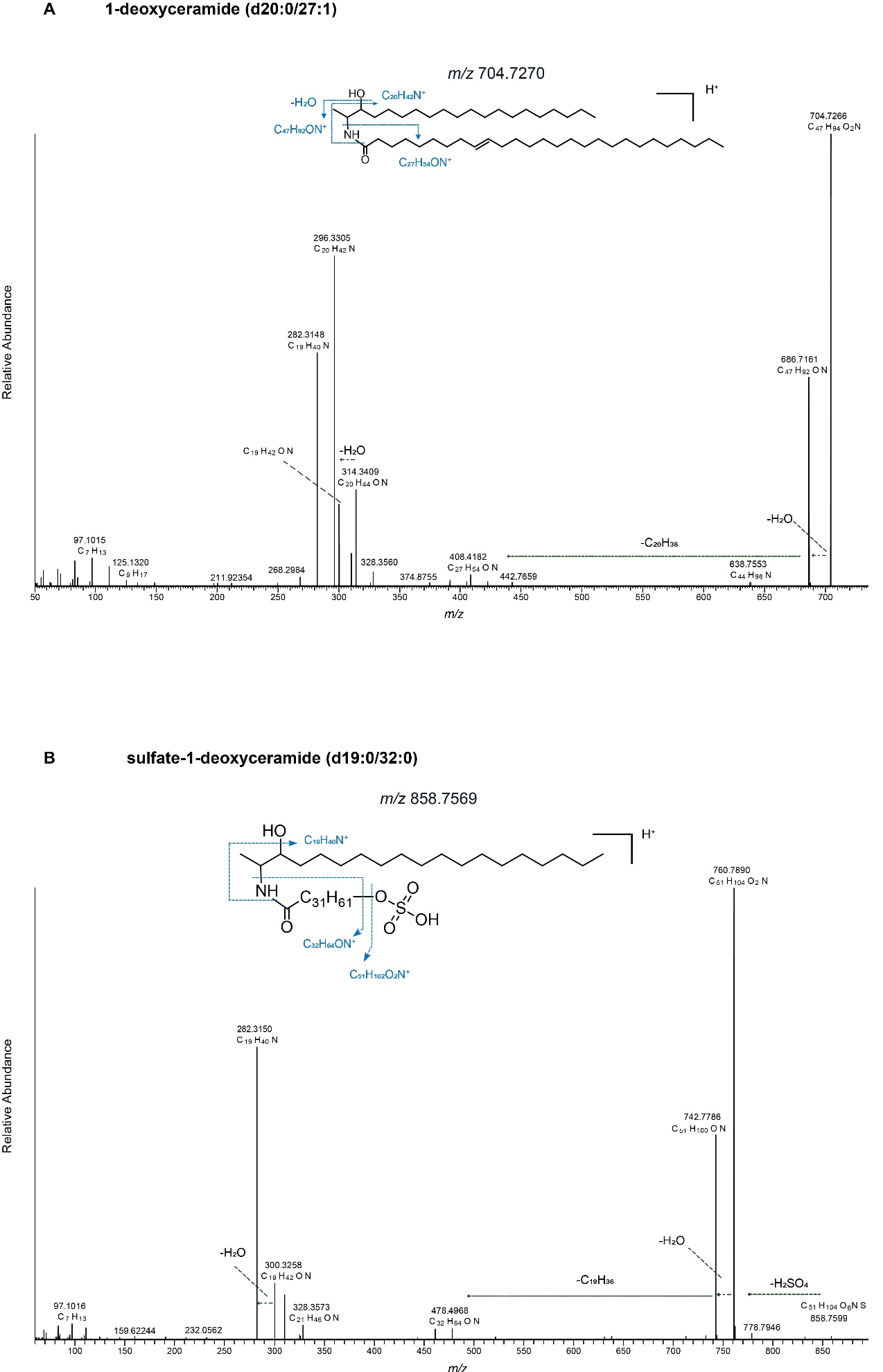


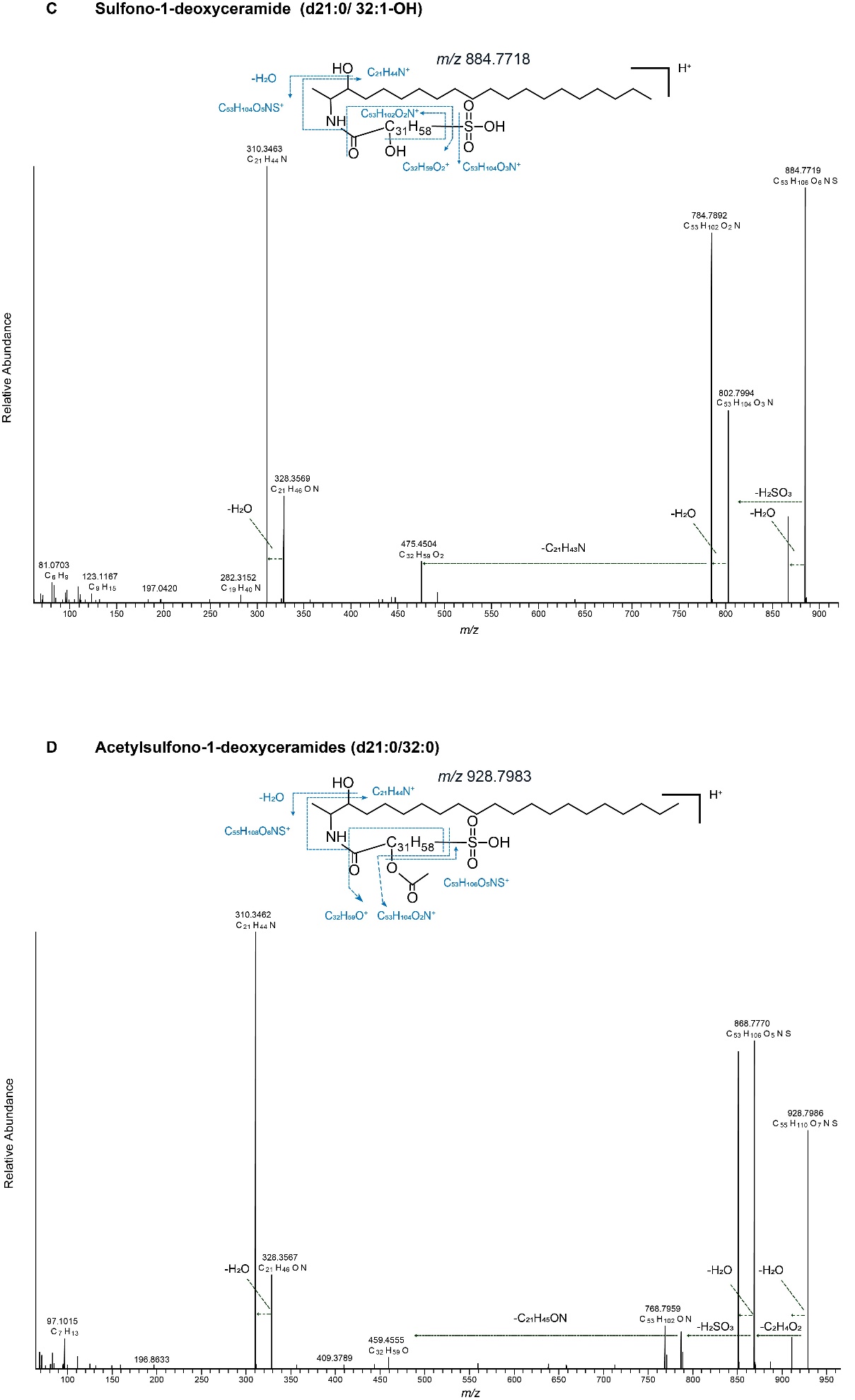


**Figure S2.** UHPLC-HRMS/MS spectra of A) 1-deoxyceramide (d20:0/27:1); B) Sulfate-1-deoxyceramide (d19:0/32:0); C) Sulfono-1-deoxyceramide (d21:0/32:1) and D) Acetylsulfono-1-deoxyceramide (d21:0/32:0).


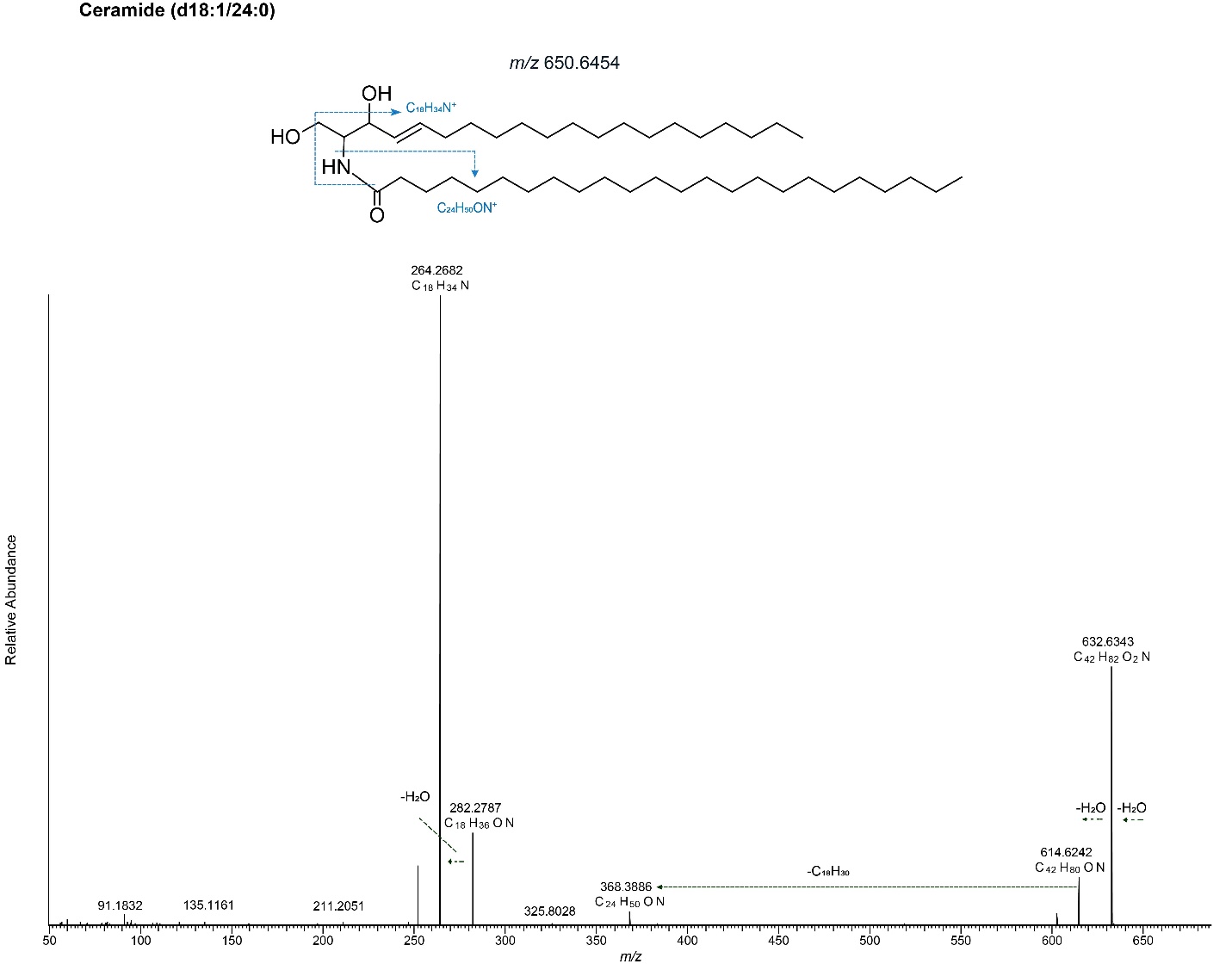


**Figure S3.** UHPLC-HRMS/MS spectra of Ceramide (d18:1/24:0).

**Table S1.** Selected abundant lipids in the Black Sea 2013. Δmmu = (Measured mass in Orbitrap – calculated mass) x 1000. AEC = Assigned elemental composition.

Lipid abbreviations: DAG, diacylglycerol; DEG, dietherglycerol; AEG, acyletherglycerol; TAG, triacylglycerol; DGTS, diacylglycerylhydroxymethyltrimethyl-(N,N,N)-homoserine; DGCC, diacylglycerylcarboxyhydroxymethylcholine; DGTA, diacylglyceryl-hydroxymethyl)-tri-methyl-b-alanine; OL, ornithine lipid; DPG, diphosphatidylglycerol; PC, phosphatidylcholine; PE, phosphatidylethanolamine; PG, phosphatidylglycerol; PI, phosphatidylinositol; MMPE, phosphatidyl-(N)-methylethanolamine; MGDG, monoglycosyldiacylglycerol. ^*^ represents the presence of a hydroxyl fatty acid as part of the lipid.

The spectral annotations are categorized in the column ‘Classification’ on the quality of the MS/MS spectra as well as the trustworthiness of the annotations. Levels marked with ^†^ indicate the lipid is annotated from the GNPS library. All other lipids are either tentatively identified in this study or have been reported in the previous studies (Schubotz et al., 2009; Van Mooy and Fredricks, 2010; Danielewicz et al., 2011; Wakeham et al., 2012; Bale et al., 2016; Kharbush et al., 2016; Becker et al., 2018b; Schubotz et al., 2018). There are three quality levels, i.e. Gold, Silver and Bronze (https://ccms-ucsd.github.io/GNPSDocumentation/spectrumcuration). Gold represents lipids that are annotated based on mass spectra derived from compounds made synthetically, identified by complete structural characterization with NMR, crystallography or other standard methods as defined in the publication guidelines for Journal of Natural Products, Privileged users. Silver represents lipids that are crude or isolated from the sample then annotated based on mass spectra interpretation. Any other putative, complete, or partial annotations are shown in level Bronze.

|  | | |  | |  | |  |  | | |  | |  |
| --- | --- | --- | --- | --- | --- | --- | --- | --- | --- | --- | --- | --- | --- |
| **Mass extracted by Mzmine (*m/z*)** | **Mass detected in Orbitrap (*m/z*)** | **Depth distribution in Black Sea (mbsl)** | **AEC** | **Δ mmu** | | **(Tentative) assignment** | | | **Classification** | | |  |  |
| DGTS/DGTA1 |  |  |  |  | |  | | | |  | |  |  |
| 804.5731 | 804.5765 | 50-90 | C_50_H_78_O_7_N^+^ | -0.8 | | DGTS (40:10) | | | | Bronze | |  |  |
| 758.5979 | 758.5915 | 50-90 | C_46_H_80_O_7_N^+^ | -1.4 | | DGTS (36:5) | | | | Bronze | |  |  |
| 740.6356 | 740.6393 | 50-90 | C_44_H_86_O_7_N^+^ | -0.6 | | DGTS (34:0) | | | | Bronze | |  |  |
| 736.6085 | 736.6076 | 50-90 | C_44_H_82_O_7_N^+^ | -1.0 | | DGTS (34:2) | | | | Bronze | |  |  |
| 732.5846 | 732.5765 | 50-90 | C_44_H_78_O_7_N^+^ | -0.8 | | DGTS (34:4) | | | | Bronze | |  |  |
| 730.5671 | 730.5598 | 50-90 | C_44_H_76_O_7_N^+^ | -1.8 | | DGTS (34:5) | | | | Bronze | |  |  |
| 682.5636 | 682.5611 | 50-90 | C_40_H_76_O_7_N^+^ | -0.6 | | DGTS (30:1) | | | | Bronze | |  |  |
| 784.6118 | 784.6077 | 50-90 | C_48_H_82_O_7_N^+^ | -0.9 | | DGTS (38:6) | | | | Bronze | |  |  |
| 762.623 | 762.6223 | 50 | C_46_H_84_O_7_N^+^ | -2.0 | | DGTS (36:3) | | | | Bronze | |  |  |
|  |  | |  |  | |  | | | |  | |  | |
| 752.6371 | 752.6396 | 130-2000 | C_45_H_86_O_7_N^+^ | -0.8 | | DGTS/DGTA (35:1) | | | | Bronze | |  |  |
| 740.6381 | 740.6385 | 130-200 | C_44_H_86_O_7_N^+^ | -2.0 | | DGTS/DGTA (34:0) | | | | Bronze | |  |  |
| 738.6216 | 738.6235 | 130-2000 | C_44_H_84_O_7_N^+^ | -1.2 | | DGTS/DGTA (34:1) | | | | Bronze | |  |  |
| 738.6229 | 738.6236 | 70-2000 | C_44_H_84_O_7_N^+^ | -1.2 | | DGTS/DGTA (34:1) | | | | Bronze | |  |  |
| 726.6225 | 726.6237 | 130-2000 | C_43_H_84_O_7_N^+^ | -1.0 | | DGTS/DGTA (33:0) | | | | Bronze | |  |  |
| 724.606 | 724.6072 | 130-2000 | C_43_H_82_O_7_N^+^ | -1.9 | | DGTS/DGTA (33:1) | | | | Bronze | |  |  |
| 724.6074 | 724.6071 | 70-200 | C_43_H_82_O_7_N^+^ | -2.1 | | DGTS/DGTA (33:1) | | | | Bronze | |  |  |
| 712.6075 | 712.6084 | 130-200 | C_42_H_82_O_7_N^+^ | -0.7 | | DGTS/DGTA (32:0) | | | | Bronze | |  |  |
| 710.5923 | 710.5933 | 50-90 | C_42_H_80_O_7_N^+^ | -0.2 | | DGTS/DGTA (32:1) | | | | Bronze | |  |  |
| 708.5733 | 708.5753 | 50-90 | C_42_H_78_O_7_N^+^ | -2.5 | | DGTS/DGTA (32:2) | | | | Bronze | |  |  |
| 698.592 | 698.5926 | 130-200 | C_41_H_80_O_7_N^+^ | -0.8 | | DGTS/DGTA (31:0) | | | | Bronze | |  |  |
| Sphingolipids |  |  |  |  | |  | | | |  | |  |  |
| 956.8299 | 956.8309 | 95-250 | C_57_H_114_O_7_NS^+^ | 2.9 | | Acetylsulfono-1-deoxyceramide (21:0/34:0) (21:0/34:1) | | | | Bronze | |  |  |
| 942.813 | 942.813 | 95-250 | C_56_H_112_O_7_NS^+^ | 0.7 | | Acetylsulfono-1-deoxyceramide (20:0/34:0) | | | | Bronze | |  |  |
| 928.7999 | 928.7994 | 95-250 | C_55_H_110_O_7_NS^+^ | 2.7 | | Acetylsulfono-1-deoxyceramide (21:0/32:0) | | | | Bronze | |  |  |
| 914.7805 | 914.783 | 95-250 | C_54_H_108_O_7_NS^+^ | 1.9 | | Acetylsulfono-1-deoxyceramide (20:0/32:0) | | | | Bronze | |  |  |
| 900.7679 | 900.7648 | 95-250 | C_53_H_106_O_7_NS^+^ | -0.6 | | Acetylsulfono-1-deoxyceramide (19:0/32:0) | | | | Bronze | |  |  |
| 884.7704 | 884.7736 | 95-250 | C_53_H_106_O_6_NS^+^ | 3.1 | | Sulfono-1-deoxyceramide (d21:0/32:1*) | | | | Bronze | |  |  |
| 870.7544 | 870.7564 | 95-250 | C_52_H_104_O_6_NS^+^ | 1.6 | | Sulfono-1-deoxyceramide (d20:0/32:1*) | | | | Bronze | |  |  |
| 856.7402 | 856.7406 | 95-250 | C_51_H_102_O_6_NS^+^ | 1.4 | | Sulfono-1-deoxyceramide (d19:0/32:1*) | | | | Bronze | |  |  |
| 928.8323 | 928.8323 | 130-250 | C_56_H_114_O_6_NS^+^ |  | | Sulfate-1-deoxyceramide (d22:0/34:0) | | | | Bronze | |  |  |
| 872.7697 | 872.7697 | 130-250 | C_52_H_106_O_6_NS^+^ | -4.0 | | Sulfate-1-deoxyceramide (d20:0/32:0) | | | | Bronze | |  |  |
| 858.7539 | 858.7539 | 130-250 | C_51_H_104_O_6_NS^+^ | -0.5 | | Sulfate-1-deoxyceramide (d19:0/32:0) | | | | Bronze | |  |  |
| 844.7383 | 844.7411 | 130-250 | C_50_H_102_O_6_NS^+^ | -1.2 | | Sulfate-1-deoxyceramide (d19:0/31:0) | | | | Bronze | |  |  |
| 596.5976 | 596.5971 | 130-2000 | C_38_H_78_O_3_N^+^ | -0.5 | | Ceramide (d21:0/17:0) | | | | Bronze | |  |  |
| 582.5812 | 582.5815 | 130-2000 | C_37_H_76_O_3_N^+^ | -0.5 | | Ceramide (d20:0/17:0) | | | | Bronze | |  |  |
| 568.5662 | 568.5659 | 130-2000 | C_36_H_74_O_3_N^+^ | -0.4 | | Ceramide (d20:0/16:0) | | | | Bronze | |  |  |
| 566.5515 | 566.5507 | 130-2000 | C_36_H_72_O_3_N^+^ | 0.0 | | Ceramide (d21:1/15:0) | | | | Bronze | |  |  |
| 564.5371 | 564.5331 | 130-2000 | C_36_H_70_O_3_N^+^ | -1.9 | | Ceramide (d19:1/17:1) | | | | Bronze | |  |  |
| 554.5495 | 554.5507 | 130-2000 | C_35_H_72_O_3_N^+^ | 0.0 | | Ceramide (d18:0/17:0) | | | | Bronze | |  |  |
| 552.534 | 552.536 | 130-2000 | C_35_H_70_O_3_N^+^ | 0.9 | | Ceramide (d20:1/15:0) | | | | Bronze | |  |  |
| 540.5342 | 540.5344 | 130-2000 | C_34_H_70_O_3_N^+^ | -0.7 | | Ceramide (d19:0/15:0) | | | | Bronze | |  |  |
| 512.5048 | 512.5029 | 130-2000 | C_32_H_66_O_3_N^+^ | -0.8 | | Ceramide (d17:0/15:0) | | | | Bronze | |  |  |
| 772.662 |  | 130-250 | C_45_H_90_O_8_N^+^ |  | | Glycosylceramide (d22:0/17:0) | | | | Bronze | |  |  |
| 758.6434 | 758.6323 | 130-250 | C_44_H_88_O_8_N^+^ | -7.0 | | Glycosylceramide (d22:0/16:0) | | | | Bronze | |  |  |
| 744.6308 |  | 130-250 | C_43_H_86_O_8_N^+^ |  | | Glycosylceramide (d21:0/16:0) | | | | Bronze | |  |  |
| 730.6115 | 730.6158 | 130-250 | C_42_H_84_O_8_N^+^ | -3.3 | | Glycosylceramide (d22:0/14:0) | | | | Bronze | |  |  |
| 746.7721 | 746.7734 | 95-250 | C_50_H_100_O_2_N^+^ | -1.4 | | 1-deoxyceramide (d21:0/29:1) | | | | Bronze | |  |  |
| 732.7579 | 732.7576 | 95-250 | C_49_H_98_O_2_N^+^ | -1.6 | | 1-deoxyceramide (d21:0/27:1) | | | | Bronze | |  |  |
| 718.7411 | 718.7423 | 95-250 | C_48_H_96_O_2_N^+^ | -1.2 | | 1-deoxyceramide (d21:0/27:1) | | | | Bronze | |  |  |
| 704.7254 | 704.7264 | 95-250 | C_47_H_94_O_2_N^+^ | -1.5 | | 1-deoxyceramide (d20:0/27:1) | | | | Bronze | |  |  |
| 690.709 | 690.7108 | 95-250 | C_46_H_92_O_2_N^+^ | -1.6 | | 1-deoxyceramide (d19:0/27:1) | | | | Bronze | |  |  |
| 682.6033 | 682.6092 | 250-1000 | C_40_H_80_O_5_N_3_^+^ | -0.6 | | Lysine-dihydroceramide (d15:0/19:1) | | | | Bronze | |  |  |
| 696.6182 | 696.6252 | 130-2000 | C_41_H_82_O_5_N_3_^+^ | -0.3 | | Lysine-dihydroceramide (d16:0/19:1) | | | | Bronze | |  |  |
| 710.6335 | 710.6387 | 130-2000 | C_42_H_84_O_5_N_3_^+^ | -2.4 | | Lysine-dihydroceramide (d17:0/19:1) | | | | Bronze | |  |  |
| 724.6524 | 724.6577 | 1000-2000 | C_43_H_86_O_5_N_3_^+^ | 1.0 | | Lysine-dihydroceramide (d18:0/19:1) | | | | Bronze | |  |  |
| PC |  |  |  |  | |  | | | |  | |  |  |
| 758.5687 | 758.5684 | 70 | C_42_H_81_O_8_NP^+^ | -1.0 | | PC-DAG (16:0/18:2) | | | | Bronze^†^ | |  |  |
| 746.6021 | 746.6051 | 70 | C_42_H_85_O_7_NP^+^ | -0.7 | | PC-AEG (16:0/18:1) | | | | Bronze^†^ | |  |  |
| 732.5511 | 732.552 | 50-70 | C_40_H_79_O_8_NP^+^ | -1.7 | | PC-DAG (14:0/18:1) | | | | Bronze^†^ | |  |  |
| 730.537 | 730.54 | 50-90 | C_40_H_77_O_8_NP^+^ | 1.9 | | PC-DAG (16:0/16:2) | | | | Bronze | |  |  |
| 718.5727 | 718.5732 | 70 | C_40_H_81_O_7_NP^+^ | -1.3 | | PC-AEG (16:0/16:1) | | | | Bronze | |  |  |
| 678.5064 | 678.5064 | 50-90 | C_36_H_73_O_8_NP^+^ | -0.4 | | PC-DAG (14:0/14:0) | | | | Bronze | |  |  |
| 676.4913 | 676.4905 | 50-90 | C_36_H_71_O_8_NP^+^ | -0.7 | | PC-DAG (14:0/14:1) | | | | Bronze | |  |  |
| 832.5821 | 832.5847 | 70 | C_48_H_83_O_8_NP^+^ | -1.0 | | PC-DAG (20:4/20:3) | | | | Bronze | |  |  |
| 830.567 | 830.5682 | 70 | C_48_H_81_O_8_NP^+^ | -1.8 | | PC-DAG (20:4/20:4) | | | | Bronze^†^ | |  |  |
| 806.5662 | 806.569 | 50-90 | C_46_H_81_O_8_NP^+^ | -1.0 | | PC-DAG (16:0/22:6) | | | | Bronze^†^ | |  |  |
| 792.5862 | 792.5893 | 70 | C_46_H_83_O_7_NP^+^ | -1.4 | | PAF (o-16:0/22:6) | | | | Bronze^†^ | |  |  |
| 778.5376 | 778.5375 | 50-90 | C_44_H_77_O_8_NP^+^ | -1.2 | | PC-DAG (18:3/18:3) | | | | Bronze^†^ | |  |  |
| 766.5734 | 766.5737 | 70 | C_44_H_81_O_7_NP^+^ | 1.4 | | PAF (o-16:0/20:5) | | | | Bronze^†^ | |  |  |
| 762.5942 | 762.5981 | 70 | C_42_H_85_O_8_NP^+^ | -3.2 | | PC-DAG (16:0/18:0) | | | | Gold† | |  |  |
| 760.5838 | 760.583 | 70 | C_42_H_83_O_8_NP^+^ | -2.6 | | PC-DAG (16:0/18:1) | | | | Bronze^†^ | |  |  |
| 756.5511 | 756.5532 | 70 | C_42_H_79_O_8_NP^+^ | -1.1 | | PC-DAG (16:0/18:3) | | | | Bronze | |  |  |
| 752.5216 | 752.5215 | 50-90 | C_42_H_75_O_8_NP^+^ | -1.5 | | PC-DAG (16:0/18:5) | | | | Bronze | |  |  |
| 744.5887 | 744.5887 | 70 | C_42_H_83_O_7_NP^+^ | -2.0 | | PAF (o-16:0/18:2) | | | | Bronze | |  |  |
| 704.5209 |  | 50-90 (absence at 70) | C_38_H_75_O_8_NP^+^ | -0.7 | | PC-DAG (30:1) | | | | Bronze | |  |  |
| 678.5061 | 678.5056 | 50-90 (absence at 70) | C_36_H_73_O_8_NP^+^ | -1.3 | | PC-DAG (28:0) | | | | Bronze | |  |  |
| PE-DAG |  |  |  |  | |  | | | |  | |  |  |
| 714.5062 | 714.5062 | 130-170 | C_39_H_73_O_8_NP^+^ | -1.7 | | PE-DAG (16:1/18:2) | | | | Bronze | |  |  |
| 702.5062 | 702.5078 | 50-90, 130-170 | C_38_H_73_O_8_NP^+^ | 0.7 | | PE-DAG (16:1/18:1) | | | | Bronze | |  |  |
| 690.5056 | 690.5081 | 70-250 | C_37_H_73_O_8_NP^+^ | 1.0 | | PE-DAG (16:0/16:1) | | | | Bronze | |  |  |
| 688.4908 | 688.4892 | 70-250 | C_37_H_71_O_8_NP^+^ | -2.5 | | PE-DAG (16:1/16:1) | | | | Bronze^†^ | |  |  |
| 676.4916 | 676.4905 | 95-250 | C_36_H_71_O_8_NP^+^ | -1.8 | | PE-DAG (15:0/16:1) | | | | Bronze | |  |  |
| 662.4759 | 662.474 | 95-250 | C_35_H_69_O_8_NP^+^ | -2.1 | | PE-DAG (14:0/16:1) | | | | Bronze | |  |  |
| PE-AEG |  |  |  |  | |  | | | |  | |  |  |
| 678.5408 | 678.5504 | 95-2000 | C_37_H_77_O_7_NP^+^ | -3.0 | | PE-AEG (o-17:0/15:0) | | | | Bronze | |  |  |
| 664.5246 |  | 95-250 | C_36_H_75_O_7_NP^+^ | -3.5 | | PE-AEG (o-16:0/15:0) | | | | Bronze | |  |  |
| 650.5117 | 650.5092 | 95-2000 | C_35_H_73_O_7_NP^+^ | -3.3 | | PE-AEG (o-15:0/15:0) | | | | Bronze | |  |  |
| 636.4956 | 636.4925 | 95-250 | C_34_H_71_O_7_NP^+^ | -4.3 | | PE-AEG (o-14:0/15:0) | | | | Bronze | |  |  |
| 704.5564 |  | 130-2000 | C_39_H_79_O_7_NP^+^ | -3.0 | | PE-AEG (o-17:0/17:1) | | | | Bronze | |  |  |
| 690.5394 | 690.5431 | 130-2000 | C_38_H_77_O_7_NP^+^ | -0.7 | | PE-AEG (o-16:0/17:1) | | | | Bronze | |  |  |
| 676.5259 | 676.5288 | 95-2000 | C_37_H_75_O_7_NP^+^ | 0.7 | | PE-AEG (o-15:0/17:1) | | | | Bronze | |  |  |
| 662.5103 | 662.513 | 95-250 | C_36_H_73_O_7_NP^+^ | 0.4 | | PE-AEG (o-15:0/16:1) | | | | Bronze | |  |  |
| 648.4954 | 648.4907 | 95-250 | C_35_H_71_O_7_NP^+^ | -6.1 | | PE-AEG (o-14:0/16:1) | | | | Bronze | |  |  |
| PE-DEG |  |  |  |  | |  | | | |  | |  |  |
| 676.5625 | 676.5618 | 250-2000 | C_38_H_79_O_6_NP^+^ | -2.7 | | PE-DEG (33:1) | | | | Bronze | |  |  |
| 674.5482 | 674.5476 | 130-2000 | C_38_H_77_O_6_NP^+^ | -1.2 | | PE-DEG (33:2) | | | | Bronze | |  |  |
| 662.5478 | 662.5472 | 105-2000 | C_37_H_77_O_6_NP^+^ | -1.7 | | PE-DEG (32:1) | | | | Bronze | |  |  |
| 660.5325 | 660.5315 | 105-2000 | C_37_H_75_O_6_NP^+^ | -1.7 | | PE-DEG (32:2) | | | | Bronze | |  |  |
| 650.5453 | 650.5472 | 105-2000 | C_36_H_77_O_6_NP^+^ | -1.7 | | PE-DEG (31:0) | | | | Bronze | |  |  |
| 648.5315 | 648.5303 | 105-2000 | C_36_H_75_O_6_NP^+^ | -2.9 | | PE-DEG (31:1) | | | | Bronze | |  |  |
| 636.5338 | 636.5322 | 105-2000 | C_35_H_75_O_6_NP^+^ | -1.0 | | PE-DEG (30:0) | | | | Bronze | |  |  |
| 634.5163 | 634.5148 | 105-170 | C_35_H_73_O_6_NP^+^ | -2.8 | | PE-DEG (30:1) | | | | Bronze | |  |  |
| MMPE/PC-DEG |  |  |  |  | |  | | | |  | |  |  |
| 704.5951 | 704.595 | 105, 130 | C_40_H_83_O_6_NP^+^ | -0.8 | | PC-DEG (32:1) | | | | Bronze | |  |  |
| 702.5796 | 702.5795 | 105, 130 | C_40_H_81_O_6_NP^+^ | -0.6 | | PC-DEG (32:2) | | | | Bronze | |  |  |
| 678.578 | 678.58 | 130-2000 | C_38_H_81_O_6_NP^+^ | -0.2 | | MMPE-DEG (32:0) | | | | Bronze | |  |  |
| 664.559 | 664.5614 | 130-2000 | C_37_H_79_O_6_NP^+^ | -3.0 | | MMPE-DEG (31:0) | | | | Bronze | |  |  |
| PG-DEG |  |  |  |  | |  | | | |  | |  |  |
| 695.5579 | 695.5561 | 105-110, 2000 | C_38_H_80_O_8_P^+^ | -3.0 | | PG-DEG (32:0) | | | | Bronze | |  |  |
| 681.5412 | 681.5409 | 95-2000 | C_37_H_78_O_8_P^+^ | -2.5 | | PG-DEG (31:0) | | | | Bronze | |  |  |
| 667.528 | 667.5259 | 95-2000 | C_36_H_76_O_8_P^+^ | -1.9 | | PG-DEG (30:0) | | | | Bronze | |  |  |
| 693.5418 | 693.5428 | 95-2000 | C_38_H_78_O_8_P^+^ | -0.6 | | PG-DEG (32:1) | | | | Bronze | |  |  |
| 691.5316 | 691.5263 | 95-200 | C_38_H_76_O_8_P^+^ | -1.5 | | PG-DEG (32:2) | | | | Bronze | |  |  |
| 665.5099 | 665.5114 | 105-170 | C_36_H_74_O_8_P^+^ | -0.7 | | PG-DEG (30:1) | | | | Bronze | |  |  |
| PI-DEG/1G-phosphate-DEG | |  |  |  | |  | | | |  | |  |  |
| 825.6189 | 825.6193 | 130-170, 2000 | C_44_H_90_O_11_P^+^ | -2.8 | | PI-DEG/1G-phosphate-DEG (35:0) | | | | Bronze | |  |  |
| 811.6035 | 811.604 | 130-250, 2000 | C_43_H_88_O_11_P^+^ | -2.4 | | PI-DEG/1G-phosphate-DEG (34:0) | | | | Bronze | |  |  |
| 797.5881 | 797.5876 | 130-250, 2000 | C_42_H_86_O_11_P^+^ | -3.1 | | PI-DEG/1G-phosphate-DEG (33:0) | | | | Bronze | |  |  |
| 783.5722 | 783.5731 | 130-250, 2000 | C_41_H_84_O_11_P^+^ | -2.0 | | PI-DEG/1G-phosphate-DEG (32:0) | | | | Bronze | |  |  |
| 769.5555 | 769.5562 | 130-250, 2000 | C_40_H_82_O_11_P^+^ | -3.2 | | PI-DEG/1G-phosphate-DEG (31:0) | | | | Bronze | |  |  |
| 755.5408 | 755.5428 | 130-250, 2000 | C_39_H_80_O_11_P^+^ | -1.0 | | PI-DEG/1G-phosphate-DEG (30:0) | | | | Bronze | |  |  |
| 1G-DEG |  |  |  |  | |  | | | |  | |  |  |
| 729.622 | 729.6233 | 130-250 | C_43_H_85_O_8_^+^ | -1.1 | | 1G-DEG (34:1) | | | | Bronze | |  |  |
| 715.6077 | 715.6077 | 130-170 | C_42_H_83_O_8_^+^ | -1.1 | | 1G-DEG (33:1) | | | | Bronze | |  |  |
| DEG |  |  |  |  | |  | | | |  | |  |  |
| 537.5225 | 537.5249 | 100-250, 2000 | C_35_H_69_O_3_^+^ | 0.3 | | DEG (16:1/16:1) | | | | Bronze | |  |  |
| 523.5073 | 523.5098 | 130-2000 | C_34_H_67_O_3_^+^ | 0.8 | | DEG (15:2/16:0) | | | | Bronze | |  |  |
| 569.586 | 569.5862 | 130-250, 1000 | C_37_H_77_O_3_^+^ | -1.1 | | DEG (17:0/17:0) | | | | Bronze | |  |  |
| 555.5582 | 555.5721 | 130-2000 | C_36_H_75_O_3_^+^ | 0.4 | | DEG (16:0/17:0) | | | | Bronze | |  |  |
| 541.5475 | 541.5538 | 130-2000 | C_35_H_73_O_3_^+^ | -2.2 | | DEG (15:0/17:0) | | | | Bronze | |  |  |
| 527.5394 | 527.542 | 130-2000 | C_34_H_71_O_3_^+^ | 1.7 | | DEG (15:0/16:0) | | | | Bronze | |  |  |
| 513.5251 | 513.5247 | 130-2000 | C_33_H_69_O_3_^+^ | 0.0 | | DEG (15:0/15:0) | | | | Bronze | |  |  |
| 539.5358 | 539.5402 | 130-2000 | C_35_H_71_O_3_^+^ | 0.0 | | DEG (16:0/16:1) | | | | Bronze | |  |  |
| 537.5235 | 537.5268 | 130-2000 | C_35_H_69_O_3_^+^ | 2.1 | | DEG (16:1/16:1) | | | | Bronze | |  |  |
| 525.5234 | 525.5223 | 130-2000 | C_34_H_69_O_3_^+^ | -2.3 | | DEG (15:0/16:1) | | | | Bronze | |  |  |
| 511.5068 | 511.5075 | 130-500 | C_33_H_67_O_3_^+^ | -1.5 | | DEG (14:0/16:1) | | | | Bronze | |  |  |
| Isorenieratene |  |  |  |  | |  | | | |  | |  |  |
| 528.3743 | 528.3734 | 95-130 | C_40_H_48_^+^ | -1.6 | | Isorenieratene | | | | Bronze | |  |  |
| β-Carotene |  |  |  |  | |  | | | |  | |  |  |
| 536.4372 | 536.4374 | 50-130 | C_40_H_56_^+^ | -0.2 | | β-Carotene | | | | Bronze | |  |  |
| Chlorophylls |  |  |  |  | |  | | | |  | |  |  |
| 905.8341 | 905.6301 | 50, 80-90 | C_60_H_81_O_3_N_4_^+^ | -0.3 | | Pyrophaeophorbide a cholesta-3ß-ol | | | | Bronze | |  |  |
| 901.8024 | 901.5978 | 50, 80-90 | C_60_H_77_O_3_N_4_^+^ | -1.2 | | Pyrophaeophorbide a cholesta-5,22-dien-3-ol | | | | Bronze | |  |  |
| 893.5393 | 893.5394 | 50-250 | C_55_H_73_O_5_N_4_Mg^+^ | -3.7 | | Chlorophyll a | | | | Bronze | |  |  |
| 891.5252 | 891.5236 | 50-90 | C_55_H_71_O_5_N_4_Mg^+^ | -3.9 | |  | | | |  | |  |  |
| 871.5711 | 871.5727 | 50-2000 | C_55_H_75_O_5_N_4_^+^ | -1.0 | | Pheophytin a | | | | Bronze | |  |  |
| 813.5666 | 813.5668 | 50-2000 | C_53_H_73_O_3_N_4_^+^ | -1.5 | | Pyropheophytin a | | | | Bronze | |  |  |
| Xanthophyll |  |  |  |  | |  | | | |  | |  |  |
| 659.429 | 659.4304 | 50-170 (absence at 70) | C_42_H_59_O_6_^+^ | -0.7 | | Fucoxanthin | | | | Bronze^†^ | |  |  |
| 641.4191 | 641.4193 | 50-170 (absence at 70) | C_42_H_57_O_5_^+^ | -1.3 | | Dinoxanthin | | | | Bronze | |  |  |
| 581.3988 | 581.398 | 50-170 (absence at 70) | C_40_H_53_O_3_^+^ | -1.5 | | Diadinoxanthin | | | | Bronze | |  |  |
| OL |  |  |  |  | |  | | | |  | |  |  |
| 683.5947 | 683.5927 | 130-170 | C_40_H_79_O_6_N_2_^+^ | -1.1 | | OH-OL (35:0) | | | | Bronze | |  |  |
| 667.5993 | 667.5983 | 130-170 | C_40_H_79_O_5_N_2_^+^ | -0.6 | | OL (35:0) | | | | Bronze | |  |  |
| 665.5827 | 665.5829 | 130-170 | C_40_H_77_O_5_N_2_^+^ | -0.3 | | OL (35:1) | | | | Bronze | |  |  |
| 653.5832 | 653.5818 | 100-250 | C_39_H_77_O_5_N_2_^+^ | -1.5 | | OL (34:0) | | | | Bronze | |  |  |
| 639.5695 | n.d. | 100-250 | C_38_H_75_O_5_N_2_^+^ | 1.9 | | OL (33:0) | | | | Bronze | |  |  |
| 625.5531 | n.d. | 100-250 | C_37_H_73_O_5_N_2_^+^ | 1.2 | | OL (32:0) | | | | Bronze | |  |  |
| Lyso PC |  |  |  |  | |  | | | |  | |  |  |
| 568.3398 | 568.3396 | 70 (50-90) | C_30_H_51_O_7_NP^+^ | -0.7 | | Lyso PC (22:6) | | | | Bronze | |  |  |
| 542.3239 | 542.3236 | 70 (50-90) | C_28_H_49_O_7_NP^+^ | -1.1 | | Lyso PC (20:5) | | | | Bronze | |  |  |
| 496.3391 | 496.3392 | 50-90 | C_24_H_51_O_7_NP^+^ | -1.1 | | Lyso PC (16:0) | | | | Gold^†^ | |  |  |
| 494.3236 | 494.3235 | 70 | C_24_H_51_O_7_NP^+^ | -1.2 | | Lyso PC (16:1) | | | | Bronze | |  |  |
| 482.3598 | 482.3596 | 70 (50-90) | C_24_H_53_O_6_NP^+^ | 1.4 | | monoether PC (16:0) | | | | Bronze^†^ | |  |  |
| AEG |  |  |  |  | |  | | | |  | |  |  |
| 599.504 | 599.5031 | 50-90 (absence at 70) | C_39_H_67_O_4_^+^ | -0.9 | | AEG(o-18:4/18:2) | | | | Bronze | |  |  |
| 575.5027 | 575.5026 | 50-90 (absence at 70) | C_37_H_67_O_4_^+^ | -1.3 | | AEG(o-18:3/16:1) | | | | Bronze | |  |  |
| 573.488 | 573.4865 | 50-90 (absence at 70) | C_37_H_65_O_4_^+^ | -1.8 | | AEG(o-18:4/16:1) | | | | Bronze | |  |  |
| 603.5337 | 603.5343 | 50-90 (absence at 70) | C_39_H_71_O_4_^+^ | -0.9 | | AEG(o-18:3/18:1) | | | | Bronze | |  |  |
| 577.5188 | 577.5182 | 50-90 | C_37_H_69_O_4_^+^ | -1.4 | | AEG(o-16:1/18:2) | | | | Bronze^†^ | |  |  |
| 577.5185 | 577.5187 | 110-170 | C_37_H_69_O_4_^+^ | -0.9 | | AEG(o-16:2/18:1) | | | | Bronze^†^ | |  |  |
| 575.5022 | 575.5026 | 50-90 (absence at 70) | C_37_H_67_O_4_^+^ | -0.8 | | AEG(o-16:2/18:2) | | | | Bronze | |  |  |
| 575.5032 | 575.502 | 50-170 | C_37_H_67_O_4_^+^ | -2.0 | | AEG(o-16:3/18:1) | | | | Bronze | |  |  |
| 551.503 | 551.5028 | 50-90 | C_35_H_67_O_4_^+^ | -1.1 | | AEG(o-16:2/16:0) | | | | Bronze | |  |  |
| 549.4888 | 549.4867 | 50-90 | C_35_H_65_O_4_^+^ | -1.6 | | AEG(o-14:1/18:2) | | | | Bronze | |  |  |
| 549.4876 | 549.4846 | 95-130 | C_35_H_65_O_4_^+^ | -1.8 | | AEG(o-16:3/16:0) | | | | Bronze | |  |  |
| 547.4713 | 547.4716 | 50-90 (absence at 70) | C_35_H_63_O_4_^+^ | -1.0 | | AEG(o-14:1/18:3) | | | | Bronze | |  |  |
| 547.4719 | 547.4718 | 50-170 | C_35_H_63_O_4_^+^ | -0.8 | | AEG(o-16:3/16:1) | | | | Bronze | |  |  |
| 535.472 | 535.4717 | 95-170 | C_34_H_63_O_4_^+^ | -1.0 | | AEG(o-16:3/15:0) | | | | Bronze | |  |  |
| 523.4717 | 523.4711 | 50-90 | C_33_H_63_O_4_^+^ | -1.5 | | AEG(o-14:1/16:1) | | | | Bronze | |  |  |
| 523.4718 | 523.471 | 95-170 | C_33_H_63_O_4_^+^ | -1.7 | | AEG(o-15:2/15:0) | | | | Bronze | |  |  |
| 521.4558 | 521.4554 | 50-90 (absence at 70) | C_33_H_61_O_4_^+^ | -1.5 | | AEG(o-14:1/16:2) | | | | Bronze | |  |  |
| 495.4409 | 495.4399 | 50-90 | C_31_H_59_O_4_^+^ | -1.4 | | AEG(o-14:1/14:1) | | | | Bronze | |  |  |
| TAG |  |  |  |  | |  | | | |  | |  |  |
| 904.8313 | 904.8322 | 50-90 (absence at 70) | C_57_H_110_O_6_N^+^ | -1.1 | | TAG (16:0/18:1/20:1)/TAG (18:0/18:1/18:1) | | | | Bronze | |  |  |
| 890.8161 | 890.8145 | 80-90 | C_56_H_108_O_6_N^+^ | -3.2 | | TAG (17:0/18:1/18:1)/TAG (16:0/18:1/19:1) | | | | Bronze^†^ | |  |  |
| 878.815 | 878.8157 | 50-90 | C_55_H_108_O_6_N^+^ | -2.0 | | TAG (16:0/18:0/18:1) | | | | Bronze | |  |  |
| 876.7986 | 876.8005 | 50-90 | C_55_H_106_O_6_N^+^ | -1.5 | | TAG (16:0/18:1/18:1) | | | | Bronze | |  |  |
| 874.7825 | 874.7839 | 50-90 | C_55_H_104_O_6_N^+^ | -2.5 | | TAG (16:0/18:1/18:2) | | | | Bronze | |  |  |
| 864.7987 | 864.8011 | 50-90 (absence at 70) | C_54_H_106_O_6_N^+^ | -0.9 | | TAG (16:0/17:0/18:1) | | | | Bronze | |  |  |
| 850.7834 | 850.784 | 50-90 | C_53_H_104_O_6_N^+^ | -2.4 | | TAG (16:0/16:0/18:1) | | | | Bronze | |  |  |
| 848.7676 | 848.767 | 50-90 | C_53_H_102_O_6_N^+^ | -3.7 | | TAG (14:0/18:1/18:1) | | | | Bronze | |  |  |
| 836.7684 | 836.7702 | 50-90 (absence at 70) | C_52_H_102_O_6_N^+^ | -0.5 | | TAG (15:0/16:0/18:1)/ TAG (16:0/16:0/17:1) | | | | Bronze | |  |  |
| 832.7369 |  | 50-90 (absence at 70) | C_52_H_98_O_6_N^+^ |  | | TAG (15:0/16:0/18:3) | | | | Bronze | |  |  |
| 808.7373 |  | 50-90 (absence at 70) | C_50_H_98_O_6_N^+^ |  | | TAG (14:0/16:0/17:1) | | | | Bronze | |  |  |
| 796.7364 | 796.7376 | 50-90 | C_49_H_98_O_6_N^+^ | -1.8 | | TAG (14:0/16:0/16:0)/ TAG (14:0/14:0/18:0) | | | | Bronze | |  |  |
| 794.7227 | 794.7205 | 50-90 | C_49_H_96_O_6_N^+^ | -3.2 | | TAG (14:0/16:0/16:1)/ TAG (14:0/14:0/18:1) | | | | Bronze | |  |  |
| 780.7055 |  | 50-90 (absence at 70) | C_48_H_94_O_6_N^+^ |  | | TAG (14:0/15:0/17:1)/ TAG (13:0/14:0/18:1) | | | | Bronze | |  |  |
| 768.706 | 768.706 | 50-90 | C_47_H_94_O_6_N^+^ | -2.1 | | TAG (14:0/14:0/16:0) | | | | Bronze | |  |  |
| 766.6905 | 766.6898 | 50-90 | C_47_H_92_O_6_N^+^ | -2.7 | | TAG (14:0/14:0/16:1) | | | | Bronze | |  |  |
| 764.6756 | 764.6769 | 50-90 (absence at 70) | C_47_H_90_O_6_N^+^ | 0.1 | | TAG (14:0/14:0/16:2) | | | | Bronze | |  |  |
| 740.6746 | 740.6741 | 50-90 (absence at 70) | C_45_H_88_O_6_N^+^ | -2.7 | | TAG (14:0/14:0/14:0) | | | | Bronze | |  |  |
| 736.6437 | 736.6426 | 90 | C_45_H_86_O_6_N^+^ | -3.0 | | TAG (12:0/12:0/18:2) | | | | Bronze | |  |  |
| 712.6438 |  | 90 | C_43_H_86_O_6_N^+^ |  | | TAG (12:0/14:0/16:0) | | | | Bronze | |  |  |
| 684.6116 | 684.612 | 90 | C_41_H_82_O_6_N^+^ | -2.2 | | TAG (12:0/12:0/14:0) | | | | Bronze | |  |  |
| 656.5813 | 656.5828 | 90 | C_39_H_78_O_6_N^+^ | -0.1 | | TAG (12:0/12:0/12:0) | | | | Bronze | |  |  |
|  |  |  |  |  | |  | | | | Bronze | |  |  |
| 1048.8298 | 1048.8299 | 50, 80, 90 | C_69_H_110_O_6_N^+^ | -2.8 | | TAG (22:2/22:6/22:6)/ TAG (16:0/22:6/28:8) | | | | Bronze | |  |  |
| 994.7826 | 994.786 | 50, 80, 90 | C_65_H_104_O_6_N^+^ | 0.2 | | TAG (18:1/22:6/22:6) | | | | Bronze | |  |  |
| 990.7562 | 990.7524 | 90 | C_65_H_100_O_6_N^+^ | -2.1 | | TAG (18:3/22:6/22:6) | | | | Bronze | |  |  |
| 988.7375 | 988.7372 | 50, 80, 90 | C_65_H_98_O_6_N^+^ | -1.6 | | TAG (18:4/22:6/22:6)/TAG (20:5/20:5/22:6) | | | | Bronze | |  |  |
| 986.7241 |  | 50, 90 | C_65_H_96_O_6_N^+^ | 0.9 | | TAG (18:5/22:6/22:6) | | | | Bronze | |  |  |
| 968.7688 | 968.7672 | 50-90 | C_63_H_102_O_6_N^+^ | -3.5 | | TAG (16:0/22:6/22:6) | | | | Bronze | |  |  |
| 966.7502 | 966.7492 | 50, 80, 90 | C_63_H_100_O_6_N^+^ | -2.7 | | TAG (18:2/20:5/22:6) | | | | Bronze | |  |  |
| 962.7204 | 962.7195 | 50-90 (absence at 70) | C_63_H_96_O_6_N^+^ |  | | TAG (18:4/20:5/22:6) | | | | Bronze | |  |  |
| 960.7066 | 960.7098 | 50-90 (absence at 70) | C_63_H_94_O_6_N^+^ | 1.7 | | TAG (18:5/20:5/22:6) | | | | Bronze | |  |  |
| 942.7516 | 942.7528 | 50-90 | C_61_H_100_O_6_N^+^ | -2.3 | | TAG (16:0/20:5/22:6) | | | | Bronze | |  |  |
| 940.7366 | 940.7353 | 50-90 | C_61_H_98_O_6_N^+^ | -2.0 | | TAG (16:1/20:5/22:6) | | | | Bronze | |  |  |
| 940.7358 | 940.737 | 50-90 (absence at 70) | C_61_H_98_O_6_N^+^ | -2.4 | | TAG (14:0/22:6/22:6) | | | | Bronze | |  |  |
| 938.7184 |  | 50-90 (absence at 70) | C_61_H_96_O_6_N^+^ | -4.8 | | TAG (18:3/18:4/22:6) | | | | Bronze | |  |  |
| 936.7039 | 936.709 | 50-90 (absence at 70) | C_61_H_94_O_6_N^+^ | 0.9 | | TAG (18:4/18:4/22:6) | | | | Bronze | |  |  |
| 934.6897 | 934.6899 | 50-90 (absence at 70) | C_61_H_92_O_6_N^+^ | -2.6 | | TAG (18:5/18:4/22:6) | | | | Bronze | |  |  |
| 916.7362 | 916.7361 | 50-90 (absence at 70) | C_59_H_98_O_6_N^+^ | -3.3 | | TAG (16:0/18:4/22:6) | | | | Bronze | |  |  |
| 914.7207 | 914.7224 | 50-90 (absence at 70) | C_59_H_96_O_6_N^+^ | -1.3 | | TAG (16:0/18:5/22:6) | | | | Bronze | |  |  |
| 912.7058 | 912.7081 | 50-90 (absence at 70) | C_59_H_94_O_6_N^+^ | 0.0 | | TAG (18:3/18:4/20:5) | | | | Bronze | |  |  |
| 910.6902 | 910.6956 | 50-90 (absence at 70) | C_59_H_92_O_6_N^+^ | 3.2 | | TAG (18:4/18:4/20:5) | | | | Bronze | |  |  |
| 908.6735 | 908.6791 | 50-90 (absence at 70) | C_59_H_90_O_6_N^+^ | 2.2 | | TAG (18:5/18:4/20:5) | | | | Bronze | |  |  |
| 884.6756 | 884.6761 | 50-90 (absence at 70) | C_57_H_90_O_6_N^+^ | -0.7 | | TAG (18:3/18:4/18:5) | | | | Bronze | |  |  |
| 882.6582 | 882.6615 | 50-90 (absence at 70) | C_57_H_88_O_6_N^+^ | 0.3 | | TAG (18:4/18:4/18:5) | | | | Bronze | |  |  |
| 880.643 | 880.6419 | 50-90 (absence at 70) | C_57_H_86_O_6_N^+^ | -3.6 | | TAG (18:5/18:4/18:5) | | | | Bronze | |  |  |
|  |  |  |  |  | |  | | | |  | |  |  |
| 1002.8446 | 1002.8445 | 50-90 | C_65_H_112_O_6_N^+^ | -3.9 | | TAG (16:0/18:1/28:8) | | | | Bronze | |  |  |
| 974.8137 | 974.8128 | 50-90 | C_63_H_108_O_6_N^+^ | -4.3 | | TAG (14:0/18:1/28:8) | | | | Bronze | |  |  |
| 972.8001 | 972.7989 | 50, 90 | C_63_H_106_O_6_N^+^ | -2.5 | | TAG (14:0/18:2/28:8) | | | | Bronze | |  |  |
| 950.8144 | 950.8145 | 50-90 | C_61_H_108_O_6_N^+^ | -2.6 | | TAG (18:0/18:1/22:6) | | | | Bronze | |  |  |
| 922.7834 | 922.7855 | 50-90 | C_59_H_104_O_6_N^+^ | -0.3 | | TAG (16:0/18:1/22:6) | | | | Bronze | |  |  |
| 920.7676 | 920.7667 | 50, 80-90 | C_59_H_102_O_6_N^+^ | -3.4 | | TAG (16:0/18:2/22:6) | | | | Bronze | |  |  |
| 910.7817 | 910.6895 | 50, 80-90 | C_59_H_92_O_6_N^+^ | -2.4 | | TAG (18:3/18:4/20:6) | | | | Bronze | |  |  |
| 894.7524 | 894.751 | 50-90 | C_57_H_100_O_6_N^+^ | -3.5 | | TAG (14:0/18:1/22:6) | | | | Bronze | |  |  |
|  |  |  |  |  | |  | | | |  | |  |  |
| 898.7807 | 898.7839 | 80-90 | C_57_H_104_O_6_N^+^ | -1.9 | | TAG (18:1/18:2/18:2) | | | | Bronze | |  |  |
| 886.783 | 886.7809 | 80-90 | C_56_H_104_O_6_N^+^ | -4.9 | | TAG (17:1/18:1/18:2) | | | | Bronze | |  |  |
| 884.7653 | 884.7676 | 90 | C_56_H_102_O_6_N^+^ | -2.6 | | TAG (17:1/18:2/18:2)/TAG (17:2/18:1/18:2) | | | | Bronze | |  |  |
| 872.7679 | 872.7668 | 50-90 | C_55_H_102_O_6_N^+^ | -3.3 | | TAG (16:0/18:2/18:2) | | | | Bronze | |  |  |
| 858.7532 |  | 85-90 | C_54_H_100_O_6_N^+^ | -1.3 | | TAG (15:0/18:2/18:2) | | | | Bronze | |  |  |
| 816.7047 | 816.707 | 50-130 | C_51_H_94_O_6_N^+^ | -0.6 | | TAG (14:0/16:0/18:4) | | | | Bronze | |  |  |
|  |  |  |  |  | |  | | | |  | |  |  |
| 926.8113 | 926.811 | 50, 80-90 | C_59_H_108_O_6_N^+^ | -6.1 | | TAG (18:2/18:2/20:1) | | | | Bronze | |  |  |
| 866.7221 | 866.7219 | 50, 80-90 | C_55_H_96_O_6_N^+^ | -1.3 | | TAG (16:0/18:3/18:4) | | | | Bronze | |  |  |
| 864.7034 | 864.7078 | 50, 80-90 | C_55_H_94_O_6_N^+^ | 0.3 | | TAG (16:0/18:4/18:4) | | | | Bronze | |  |  |
| 858.7518 | 858.7518 | 90 | C_54_H_100_O_6_N^+^ | -0.9 | | TAG (16:0/17:1/18:3)/TAG (15:0/18:1/18:3) | | | | Bronze | |  |  |
| 838.6922 | 838.6903 | 50, 80-90 | C_53_H_92_O_6_N^+^ | -1.6 | | TAG (16:0/16:3/18:4)/TAG (16:0/16:4/18:3) | | | | Bronze | |  |  |
| 836.6727 | 836.6748 | 50, 80-90 | C_53_H_90_O_6_N^+^ | -1.5 | | TAG (16:0/16:4/18:4) | | | | Bronze | |  |  |
|  |  |  |  |  | |  | | | |  | |  |  |
| 924.7944 | 924.7983 | 50-90 | C_59_H_106_O_6_N^+^ | -3.1 | | TAG (16:0/18:0/22:6) | | | | Bronze | |  |  |
| 868.7384 | 868.7334 | 50-90 | C_55_H_98_O_6_N^+^ | -5.5 | | TAG (14:0/16:0/22:6) | | | | Bronze | |  |  |
|  |  |  |  |  | |  | | | |  | |  |  |
| 820.7367 | 820.7354 | 50-130 | C_51_H_98_O_6_N^+^ | -3.5 | | TAG (14:0/16:1/18:1) | | | | Bronze | |  |  |
| 792.707 | 792.704 | 50-130 | C_49_H_94_O_6_N^+^ | -3.6 | | TAG (14:0/16:1/16:1) | | | | Bronze | |  |  |
| 790.6903 | 790.6893 | 50-90 | C_49_H_92_O_6_N^+^ | -2.6 | | TAG (14:0/16:1/16:2) | | | | Bronze | |  |  |
| DGCC |  |  |  |  | |  | | | |  | |  |  |
| 846.5861 | 846.5866 | 50-110 | C_52_H_80_O_8_N^+^ | -1.8 | | DGCC (42:11) | | | | Bronze | |  |  |
| 826.6189 | 826.6164 | 90 | C_50_H_84_O_8_N^+^ | -3.3 | | DGCC (40:7) | | | | Bronze | |  |  |
| 800.6031 | 800.6008 | 50-90 | C_48_H_82_O_8_N^+^ | -3.2 | | DGCC (38:6) | | | | Bronze | |  |  |
| 798.5911 | 798.5876 | 50-110 | C_48_H_80_O_8_N^+^ | -0.8 | | DGCC (38:7) | | | | Bronze | |  |  |
| 774.5867 | 774.5864 | 50-90 (90) | C_46_H_80_O_8_N^+^ | -2.0 | | DGCC (36:5) | | | | Bronze | |  |  |
| 746.5611 | 746.5546 | 50-90 (90) | C_44_H_76_O_8_N^+^ | -2.5 | | DGCC (34:5) | | | | Bronze | |  |  |
| 642.425 | 642.4356 | 50-90 | C_38_H_60_O_7_N^+^ | -1.4 | | DGCC (28:7) | | | | Bronze | |  |  |
| 562.3741 | 562.3731 | 50-110 | C_32_H_52_O_7_N^+^ | -1.3 | | Lyso DGCC (22:6) | | | | Bronze | |  |  |
| 536.359 | 536.3574 | 50-110 | C_30_H_50_O_7_N^+^ | -1.3 | | Lyso DGCC (20:5) | | | | Bronze | |  |  |
| 490.373 | 490.3726 | 50-90 (90) | C_26_H_52_O_7_N^+^ | -1.8 | | Lyso DGCC (18:0) | | | | Bronze | |  |  |
| 462.3419 | 462.3413 | 50-90 (90) | C_24_H_48_O_7_N^+^ | -1.8 | | Lyso DGCC (16:0) | | | | Bronze | |  |  |
| MGDG |  |  |  |  | |  | | | |  | |  |  |
| 744.5566 | 744.5842 | 50-100 | C_41_H_78_O_10_N^+^ |  | | MGDG (14:0/18:2) | | | | Bronze | |  |  |
| 742.5389 |  | 50 | C_41_H_76_O_10_N^+^ |  | | MGDG (14:0/18:3) | | | | Bronze | |  |  |
| 740.5263 | 740.5816 | 50 | C_41_H_74_O_10_N^+^ |  | | MGDG (14:0/18:4) | | | | Bronze | |  |  |
| 718.5388 | 718.5464 | 50-170 | C_39_H_76_O_10_N^+^ |  | | MGDG (14:0/16:1) | | | | Bronze | |  |  |
| 716.5246 | 716.5518 | 50, 80-90 | C_39_H_74_O_10_N^+^ |  | | MGDG (14:1/16:1) | | | | Bronze | |  |  |
| 788.5264 |  | 50-90 (absence at 70) | C_45_H_74_O_10_N^+^ | -4.3 | | MGDG (18:4/18:4)/MGDG (16:3/20:5) | | | | Bronze | |  |  |
| 786.5137 |  | 50-90 (absence at 70) | C_45_H_72_O_10_N^+^ | -1.4 | | MGDG (18:4/18:5) | | | | Bronze | |  |  |
| DAG |  |  |  |  | |  | | | |  | |  |  |
| 684.553 |  | 50-90 | C_43_H_74_O_5_N^+^ | -3.2 | | DAG (18:1/22:6) | | | | Bronze | |  |  |
| 658.5402 |  | 50-90 | C_41_H_72_O_5_N^+^ | -0.3 | | DAG (16:0/22:6) | | | | Bronze | |  |  |
| 656.5261 |  | 50-90 (absence at 70) | C_41_H_70_O_5_N^+^ | 1.2 | | DAG (16:1/22:6) | | | | Bronze | |  |  |
| 634.5391 |  | 50-90 (absence at 70) | C_39_H_72_O_5_N^+^ | -1.4 | | DAG (18:1/18:3)/ DAG (18:2/18:2) | | | | Bronze | |  |  |
| 632.5247 |  | 50-90 (absence at 70) | C_39_H_70_O_5_N^+^ | -0.2 | | DAG (16:0/20:5) | | | | Bronze | |  |  |
| 630.5097 |  | 50-90 | C_39_H_68_O_5_N^+^ | 0.5 | | DAG (14:0/22:6) | | | | Bronze | |  |  |
| 628.4948 |  | 50-90 (absence at 70) | C_39_H_66_O_5_N^+^ | 1.2 | | DAG (18:3/18:4)/DAG (16:2/20:5) | | | | Bronze | |  |  |
| 612.5544 |  | 50-90 | C_37_H_74_O_5_N^+^ | -1.8 | | DAG (16:0/18:1) | | | | Bronze | |  |  |
| 610.5393 |  | 50-90 (absence at 70) | C_37_H_72_O_5_N^+^ | -1.1 | | DAG (16:0/18:2) | | | | Bronze | |  |  |
| 608.5258 |  | 50-90 (absence at 70) | C_37_H_70_O_5_N^+^ | 0.9 | | DAG (16:0/18:3) | | | | Bronze | |  |  |
| 606.5098 |  | 50-90 (absence at 70) | C_37_H_68_O_5_N^+^ | 0.6 | | DAG (16:0/18:4) | | | | Bronze | |  |  |
| 604.4939 |  | 50-90 (absence at 70) | C_37_H_66_O_5_N^+^ | 0.3 | | DAG (16:0/18:5) | | | | Bronze | |  |  |
| 586.5397 |  | 50-90 (absence at 70) | C_35_H_72_O_5_N^+^ | -0.8 | | DAG (16:0/16:0) | | | | Bronze | |  |  |
| 582.5085 |  | 50-90 (absence at 70) | C_35_H_68_O_5_N^+^ | -0.7 | | DAG (14:0/18:2) | | | | Bronze | |  |  |
| 556.4931 |  | 50-90 (absence at 70) | C_33_H_66_O_5_N^+^ | -0.5 | | DAG (14:0/16:1) | | | | Bronze | |  |  |
| 626.4775 |  | 50-90 (absence at 70) | C_39_H_61_O_5_N^+^ | -0.4 | | DAG (36:9) | | | | Bronze | |  |  |
| 624.4613 |  | 50-90 (absence at 70) | C_39_H_59_O_5_N^+^ | -1.0 | | DAG (36:10) | | | | Bronze | |  |  |

**Table S2.** Most specific lipids and their S_ij_ index (top 3) throughout the water column of the Black Sea (50 to 2000 mbsl).

| Depth (m) | Lipid annotation (specificity score, S_ij_) | | | | | |
| --- | --- | --- | --- | --- | --- | --- |
|  | Lipid 1 | S_ij_ | Lipid 2 | S_ij_ | Lipid 3 | S_ij_ |
| 50 | DGTS (36:3) | 38.5 | MGDG (14:0/18:4) | 38.2 | DGTS (34:0) | 25.3 |
| 70 | PAF (o-16:0/22:6) | 28.4 | Lyso PC (20:5) | 27.6 | PC-AEG (16:0/18:1) | 27.4 |
| 80 | TAG (18:3/18:4/20:6) | 11.1 | Pyrophaeophorbide a cholesta-3ß-ol | 8.9 | DAG (16:0/20:5) | 7.9 |
| 85 | TAG (16:0/17:1/18:3)/TAG (15:0/18:1/18:3) | 45.2 | TAG (17:1/18:1/18:2) | 13.4 | TAG (18:3/18:4/20:6) | 12.4 |
| 90 | TAG (18:3/22:6/22:6) | 25.6 | TAG (17:1/18:2/18:2)/TAG (17:2/18:1/18:2) | 22.5 | TAG (18:1/18:2/18:2) | 19.0 |
| 95 | Isorenieratene | 12.2 | AEG(o-14:1/16:2) | 9.5 | Lyso DGCC (20:5) | 7.1 |
| 100 | MGDG (14:0/18:2) | 8.0 | TAG (14:0/15:0/17:1)/ TAG (13:0/14:0/18:1) | 6.1 | DAG (14:0/18:2) | 4.3 |
| 105 | DGCC (28:7) | 13.6 | PC-DEG (32:2) | 4.3 | PAF (o-16:0/18:2) | 4.0 |
| 110 | PC-DEG (32:2) | 5.5 | PE-DAG (14:0/16:1) | 4.3 | Isorenieratene | 4.2 |
| 130 | Sulfate-1-deoxyceramide (d22:0/34:0) | 11.0 | OL (35:1) | 5.2 | 1G-DEG (34:1) | 4.6 |
| 170 | 1G-DEG (34:1) | 12.7 | 1G-DEG (33:1) | 10.9 | Sulfate-1-deoxyceramide (d22:0/34:0) | 9.7 |
| 250 | OL (35:0) | 7.9 | PE-AEG (o-16:0/15:0) | 7.5 | Acetylsulfono-1-deoxyceramide (d21:0/32:0) | 6.2 |
| 500 | DEG (15:0/17:0) | 7.4 | Ceramide (d17:0/20:0) | 6.4 | OH-OL (35:0) | 6.2 |
| 1000 | PE-DEG (33:1) | 18.8 | Lysine-dihydroceramide (d15:0/22:1) | 17.8 | Lysine-dihydroceramide (d15:0/19:1) | 12.2 |
| 2000 | PC-DAG (16:0/18:0) | 15.6 | DGTS/DGTA (34:1) | 13.9 | DGTS/DGTA (35:1) | 12.1 |
